# KMT1 family methyltransferases regulate heterochromatin–nuclear periphery tethering via histone and non‐histone protein methylation

**DOI:** 10.15252/embr.201643260

**Published:** 2019-03-12

**Authors:** Radhika Arasala Rao, Alhad Ashok Ketkar, Neelam Kedia, Vignesh K Krishnamoorthy, Vairavan Lakshmanan, Pankaj Kumar, Abhishek Mohanty, Shilpa Dilip Kumar, Sufi O Raja, Akash Gulyani, Chandra Prakash Chaturvedi, Marjorie Brand, Dasaradhi Palakodeti, Shravanti Rampalli

**Affiliations:** ^1^ Centre For Inflammation and Tissue Homeostasis Institute for Stem Cell Biology and Regenerative Medicine (inStem) Bangalore Karnataka India; ^2^ Sastra University Tirumalaisamudram, Thanjavur Tamilnadu India; ^3^ Technologies for the Advancement of Science Institute for Stem Cell Biology and Regenerative Medicine (inStem) Bangalore Karnataka India; ^4^ Department of Hematology Sanjay Gandhi Postgraduate Institute of Medical Sciences Lucknow Uttar Pradesh India; ^5^ Sprott Centre for Stem Cell Research Ottawa Hospital Research Institute Ottawa ON Canada

**Keywords:** aging, euchromatic histone methyltransferases, heterochromatin, LaminB1, nuclear periphery, Chromatin, Epigenetics, Genomics & Functional Genomics, Post-translational Modifications, Proteolysis & Proteomics

## Abstract

Euchromatic histone methyltransferases (EHMTs), members of the KMT1 family, methylate histone and non‐histone proteins. Here, we uncover a novel role for EHMTs in regulating heterochromatin anchorage to the nuclear periphery (NP) via non‐histone methylation. We show that EHMTs methylate and stabilize LaminB1 (LMNB1), which associates with the H3K9me2‐marked peripheral heterochromatin. Loss of LMNB1 methylation or EHMTs abrogates heterochromatin anchorage at the NP. We further demonstrate that the loss of EHMTs induces many hallmarks of aging including global reduction of H3K27methyl marks and altered nuclear morphology. Consistent with this, we observe a gradual depletion of EHMTs, which correlates with loss of methylated LMNB1 and peripheral heterochromatin in aging human fibroblasts. Restoration of EHMT expression reverts peripheral heterochromatin defects in aged cells. Collectively, our work elucidates a new mechanism by which EHMTs regulate heterochromatin domain organization and reveals their impact on fundamental changes associated with the intrinsic aging process.

## Graphical abstract

EHMTs methylate LMNB1 to regulate its stability and localization and to promote heterochromatin assembly at the nuclear periphery. Depletion of EHMTs during physiological aging results in loss of heterochromatin organization in aged cells.

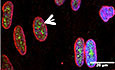

## Introduction

The Euchromatic histone lysine methyltransferases G9a, encoded by EHMT2, and GLP, encoded by EHMT1 (KMT1/Suv39 methyltransferase family), are present as heteromeric complex and negatively regulate gene transcription. The SET domain of EHMT catalyzes mono‐ and dimethylation of lysine residues at histone3 (H3) *in vitro* and *in vivo* 1, 2, 3, 4, 5. H3K9me2 deposited by EHMT1/2 complex demarcates heterochromatin, particularly non‐genic regions, and is prevalent in gene deserts, pericentromeric and subtelomeric regions, with little being observed at individual active or silent genes. Non‐coding and gene containing DNA present at the NP are also marked by the presence of H3K9me2 which spans several megabases 6, 7. Specifically, these domains are strongly correlated with binding of LMNB1 and are depleted of H3K4me3 and RNA polymerase II activity 6. These data suggest that H3K9me2 domains are critical determinants of higher‐order chromosome structure in association with the nuclear lamina (NL).

In mammalian cells, the NL acts as a hub for multiple cellular functions including chromatin organization 8, 9, 10, 11. NL is composed of A‐ and B‐type lamins along with inner nuclear membrane (INM) proteins 12, and together with mediator proteins such as barrier‐to‐autointegration factor (BAF) and heterochromatin protein 1 (HP1), facilitate attachment of chromatin to NL 13, 14. Additionally, these interactions have been proposed to form specific chromatin organization that opposes transcriptional activity 15. The association between LMNB receptor and LMNA/C mediates peripheral heterochromatin attachment in a wide variety of mammalian cells 16. Any perturbation in such organization leads to a complete loss of peripheral heterochromatin and developmental abnormalities 17.

Recent studies demonstrated that the lamina‐associated domains (LADs) enriched in H3K9 methyl (me2/me3) marks contact NL via association with LMNB1 7, 18, 19, 20. These interactions are highly stochastic in nature and are dependent on H3K9me2 activity governed by G9a/EHMT2. Accordingly, G9a/EHMT2 promotes LAD formation and its loss leads to the opposite effect 18. Similar to humans, H3K9 methylation is important for heterochromatin positioning in *C. elegans* 21, as depletion of H3K9 methyltransferases Met2 and Set‐25 (mammalian SETDB1 and G9a/EHMT2 homolog) leads to detachment of large gene array from peripheral heterochromatin. Altogether, loss of lamins and INM proteins or H3K9me2 activity leads to peripheral heterochromatin defects, however, whether there is a link between these common consequences remains unknown. Here, we establish EHMT proteins as a common module that governs heterochromatin tethering via histone‐dependent (H3K9me2) and histone‐independent mechanisms (by directly regulating LMNB1 methylation).

## Results

### EHMTs associates with LMNB1

**Figure 1:**
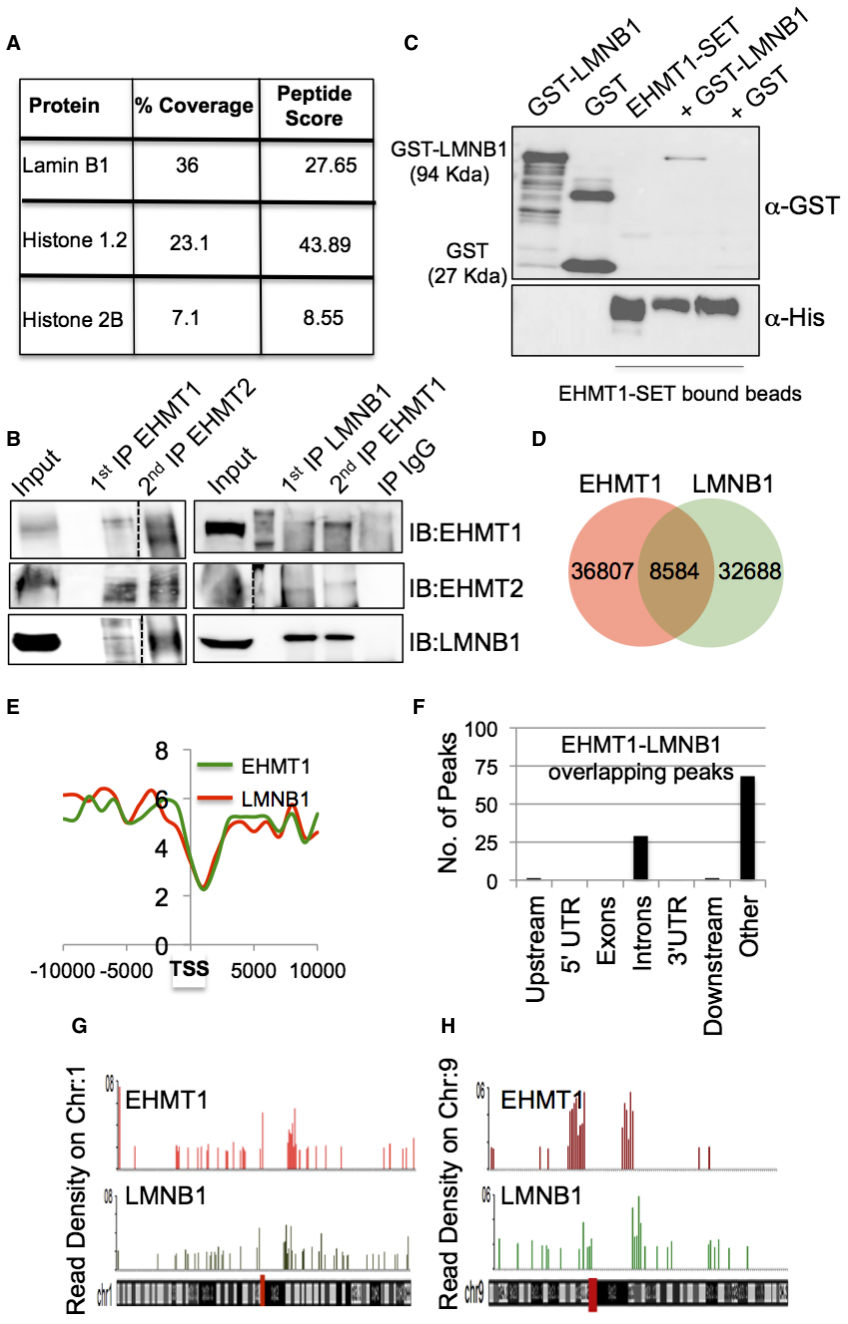
EHMT1, EHMT2, and LMNB1 are members of the same complex AEHMT1 interacting proteins identified by mass spectrometric analysis with details indicating coverage and peptide score.BSequential IP in HEK293 cells demonstrating EHMT1, EHMT2, and LMNB1 are a part of the same complex. The dotted line indicates spliced lane from two different exposures of the same gel (source data file linked).CLMNB1 interacts with EHMT1 via SET domain. Recombinant GST or GST‐LMNB1 was incubated with Ni‐NTA bound His‐EHMT1 SET protein. Postwashing eluents were loaded for immunoblotting using GST or His antibody. Recombinant pure proteins GST‐LMNB1 (lane 1), GST (lane 2), and EHMT1‐SET (lane 3) were used as controls.DVenn diagram showing unique and overlapping reads obtained from EHMT1 and LMNB1 ChIP sequencing.EComposite profile of EHMT1 and LMNB1 read density around the transcription start site (TSS).FGenomic distribution of EHMT1 and LMNB1 peaks. The majority of binding sites obtained were enriched in an intronic region or distal regions from a gene.G, HRepresentative figure showing normalized ChIP‐seq read density (above 1.5‐fold over expected) of EHMT1 and LMNB1 in 1 MB bin for chromosomes 1 and 9. Source data are available online for this figure.

**Figure EV1:**
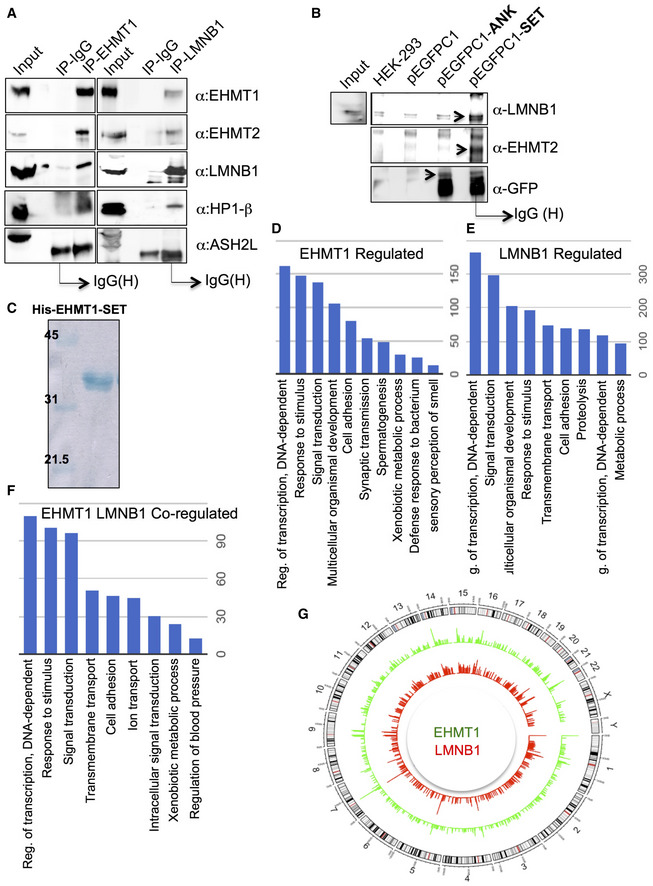
EHMT1 interacts with LMNB1 domain and co‐regulate a subset of genes AEHMT1 interacts with EHMT2, LMNB1, and HP1. Whole cell lysates from fetal HDFs were subjected to IP reaction using EHMT1 or LMNB1 antibody. Subsequently, Western blot was performed using the IPed material to detect EHMT1, EHMT2, LMNB1, and HP1. ASH2L did not show any interaction with EHMT1 and LMNB1 thus acted as a negative control.BEHMT1 interacts with LMNB1 and EHMT2 via SET domain. HEK293 cells were transfected with pEGFP‐Ankyrin (pEGFPC1‐ANK) or pEGFPC1‐SET domains of EHMT1 to determine domain‐specific association with LMNB1. Cell extracts were subjected to IP using GFP‐antibody, and the bound complexes were then analyzed by immunoblotting using LMNB1, EHMT2, and GFP antibodies. HEK‐293 whole cell extract represents 1% input. pEGFPC1 empty vector transfected HEK293 or untransfected HEK293 cells were used as control reactions. Arrows indicate specific band.CCoomassie‐stained SDS–PAGE gel showing 6X His EHMT1‐SET purified protein used for methyltransferase assays.D–FGene ontology (GO) analysis of EHMT1 and LMNB1 bound genes. Representative figure showing enriched GO terms for EHMT1 (D), LMNB1 (E), and EHMT1 and LMNB1 (F) co‐bound genes. The length of the bar (*y*‐axis) denotes total genes falling within GO term.GCircos plot showing genome wide peak density of EHMT1 (green) and LMNB1 (red).

To identify the novel non‐histone interactors of EHMT proteins, endogenous EHMT1 was immunoprecipitated (IP) and unique bands in the EHMT1 pull down were subjected to LC/MS analysis. We found LMNB1 and histone proteins as interactors of EHMT1 (Fig 1A). Mass spectrometry data were validated by sequential IP reactions of endogenous EHMT1 and LMNB1 proteins (Fig 1B). This interaction was also found using nuclear extracts from human dermal fibroblasts (HDFs) (Fetal derived unless mentioned otherwise), suggesting that it is not cell type‐specific interaction (Fig EV1A). Consistent with previously published reports, we also detected HP1 in association with EHMT and LMNB1 (Fig EV1A). The absence of Ash2 l (a member of H3K4 methyltransferase complex) in the IP‐EHMT1 or IP‐LMNB1 confirmed the specificity of IP reaction (Fig EV1A). Mapping experiments (*in vivo* and *in vitro)* to identify the LMNB1 interacting domain of EHMT1, revealed that the SET domain of EHMT1 is sufficient to bind to LMNB1 (Figs EV1B and C, and 1C). These results confirmed that the EHMT1/2 directly associates with LMNB1 via its SET domain.

Both EHMT and LMNB1 are known to interact with chromatin independently or via mediator proteins 17, 22, 23. To identify EHMT1‐LMNB1 co‐bound regions in the genome, we performed ChIP‐seq analysis. Individually, EHMT1 and LMNB1 occupied 36807 and 32688 number of peaks, respectively, and 8584 peaks were co‐bound by EHMT1 and LMNB1 (Fig 1D). A majority of EHMT1 and LMNB1 reads were distributed on non‐TSS regions such as introns and gene‐poor regions (represented as “others”) (Fig 1E and F), whereas only 1.5% of reads were found on the upstream region of genes (Fig 1F). Functional category analysis of the genes occupied by EHMT1, LMNB1, or EHMT1‐LMNB1 revealed enrichment of genes regulating transcription, signal transduction, and cell adhesion (Fig EV1D–F).

H3K9me2 deposited by EHMT1/2 complex demarcates heterochromatin, particularly non‐genic regions and is prevalent in “other regions” such as gene deserts, pericentromeric and subtelomeric regions, with little being observed at individual active or silent genes. Detailed analysis of read densities performed on individual chromosomes identified a significant correlation (*P* < 0.001) between EHMT1 and LMNB1 localization. Interestingly, we observed higher ChIP‐seq read density onto subtelomeric, and around centromeric regions (indicated as a red line on the chromosome) that are preferentially maintained in the silent state (Figs 1G and H, and EV1G). These data suggested that EHMT1‐LMNB1 associates on gene‐poor areas that are the critical determinants of higher‐order chromosome structure at the NP.

### EHMTs methylate LMNB1

**Figure EV2:**
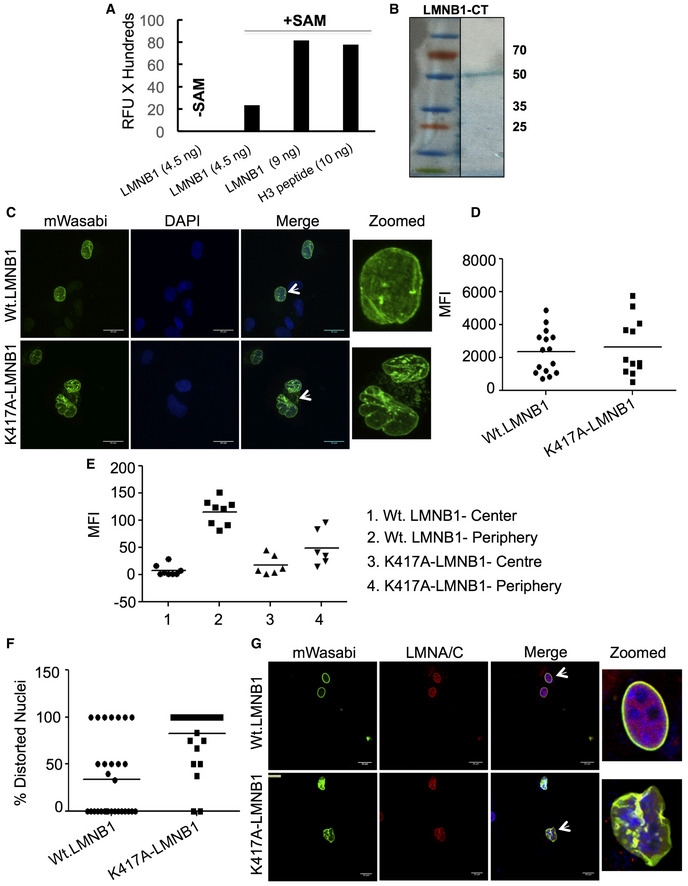
Mutation in LMNB1 causes distortion of the nuclear architecture Increasing concentrations of LMNB1‐GST showed a greater degree of methylation by EHMT1‐SET. Methyltransferase assay was performed using a fixed concentration of recombinant 6X His EHMT1‐SET as an enzyme source and SAM as a methyl group donor. Recombinant GST‐LMNB1 (4.5 ng and 9 ng) and Histone H3 peptide (10 ng) were used as substrates in the assay (*n* = 2). Histone H3 peptide was used as a positive control. The mean relative fluorescence unit (RFU) values (*n* = 1) represented in the graph were obtained after subtracting the values for controls EHMT1‐SET only, SAM only, LMNB1‐GST (4.5 ng) with those of the LMNB1‐GST (4.5 ng and 9 ng) and Histone H3 (10 ng).Coomassie‐stained SDS–PAGE gel showing 6X His LMNB1‐CT purified protein used for methyltransferase assays.mWasabi expression in fetal HDFs transduced with Wt.LMNB1 and K417A‐LMNB1 mutant construct (scale bar: 20 μm). Arrows indicate the cells zoomed in the far‐right image presented.MFI for mWasabi expression in cells transfected with Wt.LMNB1 and K417A‐LMNB1 mutant constructs. Each biological replicate (*n* = 2) is represented as spread of technical replicates around the mean value.MFI for mWasabi expression in cells transfected with Wt.LMNB1 and K417A‐LMNB1 mutant constructs. MFI has been represented as center versus periphery of the nuclei. Individual biological replicates (*n* = 2) are plotted with the mean values.Quantitation for percentage distorted nuclei in cells transfected with K417A‐LMNB1 mutant construct compared to Wt.LMNB1 construct. Individual biological replicates (*n* = 2) are plotted with the median.Immunostaining for LMNA/C in fetal HDFs transduced with Wt.LMNB1 and K417A‐LMNB1 mutant construct (scale bar: 20 μm). Arrows indicate the cells zoomed in the far‐right image presented.

**Figure 2:**
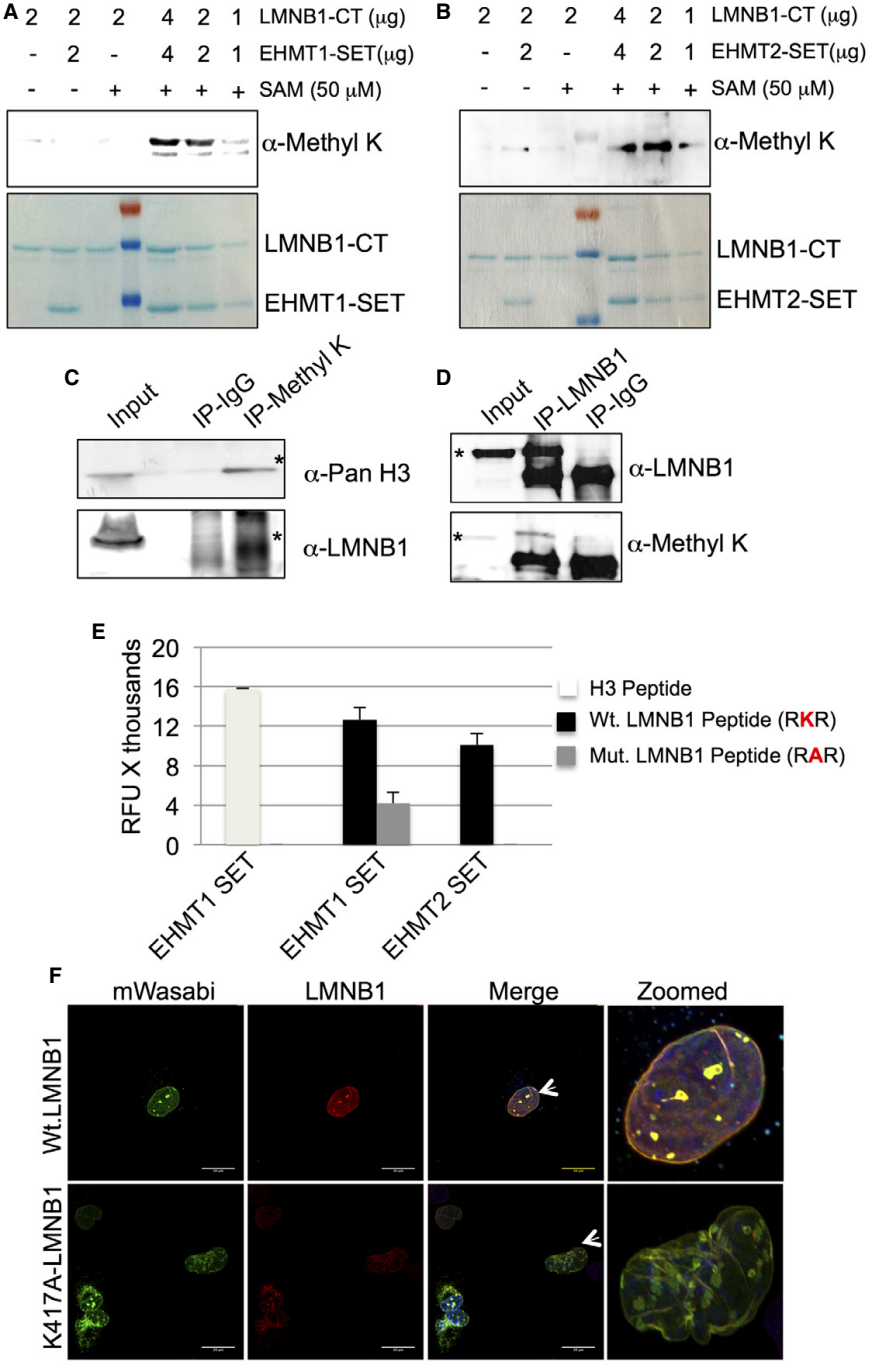
EHMT1 and EHMT2 methylate LMNB1 at C‐terminus A, BWestern blot probed with the anti‐Methyl‐K antibody. Lanes to the left of the ladder are control reactions containing 2 μg LMNB1‐CT + 50 μM SAM (lane 1) or 2 μg of EHMT1‐SET/EHMT2‐SET + 2 μg LMNB1‐CT (lane 2) and 2 μg LMNB1‐CT only (lane 3). Lanes to the right of the ladder are methyltransferase reactions containing 1, 2, and 4 μg of LMNB1‐CT and EHMT1‐SET/EHMT2‐SET along with 50 μM SAM. Lower panel: Coomassie‐stained gel representing all the reactions mentioned above.C, DNuclear lysates from HEK‐293 were immunoprecipitated with anti‐Methyl‐K or anti‐LMNB1 antibody followed by Western blotting with anti‐Pan H3, anti‐LMNB1, and anti‐Methyl‐K antibodies. Asterisks indicate the bands of interest.EReduced methylation of LMNB1 by EHMT1 and EHMT2 upon single amino acid substitution from lysine (RKR) to alanine (RAR). Mutant‐LMNB1 peptides were synthesized and subjected to methyltransferase reaction containing EHMT1‐SET and EHMT2‐SET with SAM as a methyl donor (*n* = 2). H3 peptide was used as a positive control for the reaction (*n* = 2). Error bars are represented as ±SD.FImmunostaining for LMNB1 in fetal HDFs transfected with Wt.LMNB1 and K417A‐LMNB1 constructs (scale bar: 20 μm). Arrows indicate the cells zoomed in the far‐right image presented.

Next, we tested if LMNB1 is a substrate for methylation by the EHMT enzymes. Using an *in vitro* fluorometric methyltransferase assay, we demonstrate an increase in fluorescence upon incubation of EHMT1‐SET domain with GST‐LMNB1 in the presence of S‐adenosyl methionine (SAM) (Fig EV2A). To confirm that EHMT proteins indeed methylate LMNB1, we used lysine methyl‐specific (Methyl‐K) antibody to probe for methylated LMNB1. Purified LMNB1 C‐terminus protein containing the rod domain and tail domains (LMNB1‐CT) (Fig EV2B) was used in this assay. Toward this, we performed *in vitro* methyltransferase assay using different concentrations of LMNB1 and incubated with an equimolar ratio of the EHMT1/2‐SET domain in the presence or absence of SAM. When products of these reactions were immunoblotted using the Methyl‐K antibody, specific methylation signal was observed upon incubation of LMNB1 with EHMT1/2‐SET in the presence of SAM (Fig 2A and B).

Further, to examine whether LMNB1 is methylated *in vivo*, we performed anti‐Methyl‐K or anti‐LMNB1 IPs from HEK nuclear lysates. Products of IPs were split into two halves and probed with either anti‐LMNB1 or anti‐Methyl‐K‐specific antibodies. Several lysine residues are methylated on the H3 tail, detecting the histone signal in IP‐Methyl‐K confirmed the specificity of IP reaction and served as a positive control (Fig 2C). The presence of a LMNB1 band in the same Methyl‐K IP samples indicated the presence of endogenous methylated LMNB1 (Fig 2C). In reciprocal IP reaction in which LMNB1 was IP'ed and probed with Methyl‐K antibody, identification of Methyl‐K signal in the IP‐LMNB1 confirmed LMNB1 methylation *in vivo* (Fig 2D).

It has been reported that EHMT2 is capable of methylating lysine on dipeptide Arg‐Lys (RK) sequence of non‐histone proteins 24. We synthesized peptides for such motifs present at the C‐terminus of LMNB1 and identified K417 as the methylation site targeted by EHMT1 and EHMT2 (Fig 2E). K417A peptide mutation abolished methylation of LMNB1 (Fig 2E). To investigate the function of methylated LMNB1 *in vivo,* we mutated the 417K residue to alanine (K417A) in the wild‐type (Wt.) LMNB1 construct. As opposed to Wt.LMNB1, which was localized at the NP, much of K417A‐LMNB1 was accumulated in the nucleoplasm (Figs 2F and EV2C–E). We also observed aggregates of mutant LMNB1 transported into the cytoplasm and was accompanied by abnormal nuclear morphology (Figs 2F and EV2C and F). Co‐staining with LMNB1 antibody showed localization of endogenous LMNB1 and overexpressed Wt.LMNB1 at the NP in Wt.LMNB1 expressing cells (Fig 2F). However, in mutant‐LMNB1 expressing HDFs, endogenous LMNB1 was localized in K417A‐LMNB1 aggregates indicating a dominant negative function of the mutant protein (Fig 2F). Further mislocalization of LMNA/C in the aggregates of mutant‐LMNB1 (Fig EV2G) indicated LMNB1 methylation is critical for maintaining the NL meshwork composition at the periphery. Based on these data, we speculated that the methylation modification prevents the degradation of LMNB1 and confers protein stability.

### EHMTs regulate LMNB1 levels

**Figure EV3:**
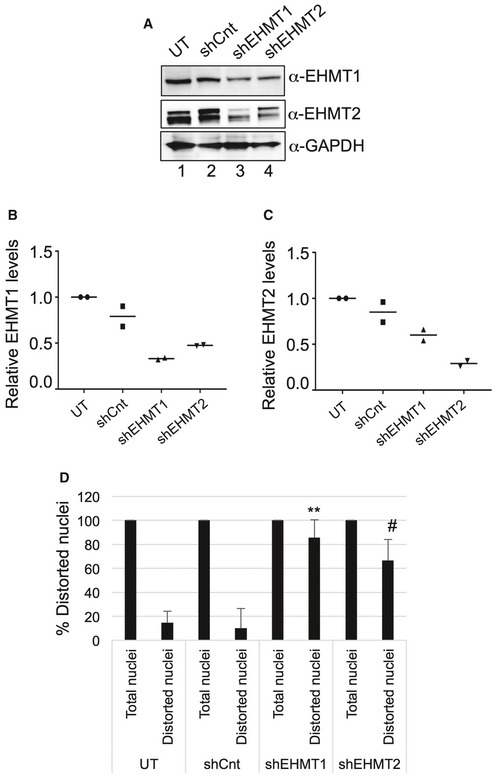
Knockdown of EHMT1 and EHMT2 causes nuclear distortion AWestern blot analysis for EHMT1 and EHMT2 in fetal HDFs upon knockdown of EHMT1 and EHMT2. GAPDH was used as a loading control.B, CRelative protein levels of EHMT1 (B) and EHMT2 (C) upon knockdown. Each biological replicate (*n* = 2) is represented as spread of technical replicates around the mean value.DPercentage of distorted nuclei increases in human fibroblasts transduced with shEHMT1 and shEHMT2 virus compared to UT or shCnt. Three biological replicates (*n* = 3) were used in this analysis. For UT, (nuclei = 43); shCnt, (nuclei = 23); shEHMT1, (nuclei = 18); shEHMT2, (nuclei = 20) nuclei counted. shCnt versus shEHMT1, **P = 0.0048; shCnt versus shEHMT2, ^#^ *P* = 0.0153 (Kruskal–Wallis test, post hoc test: Dunn's multiple comparison test).

**Figure 3:**
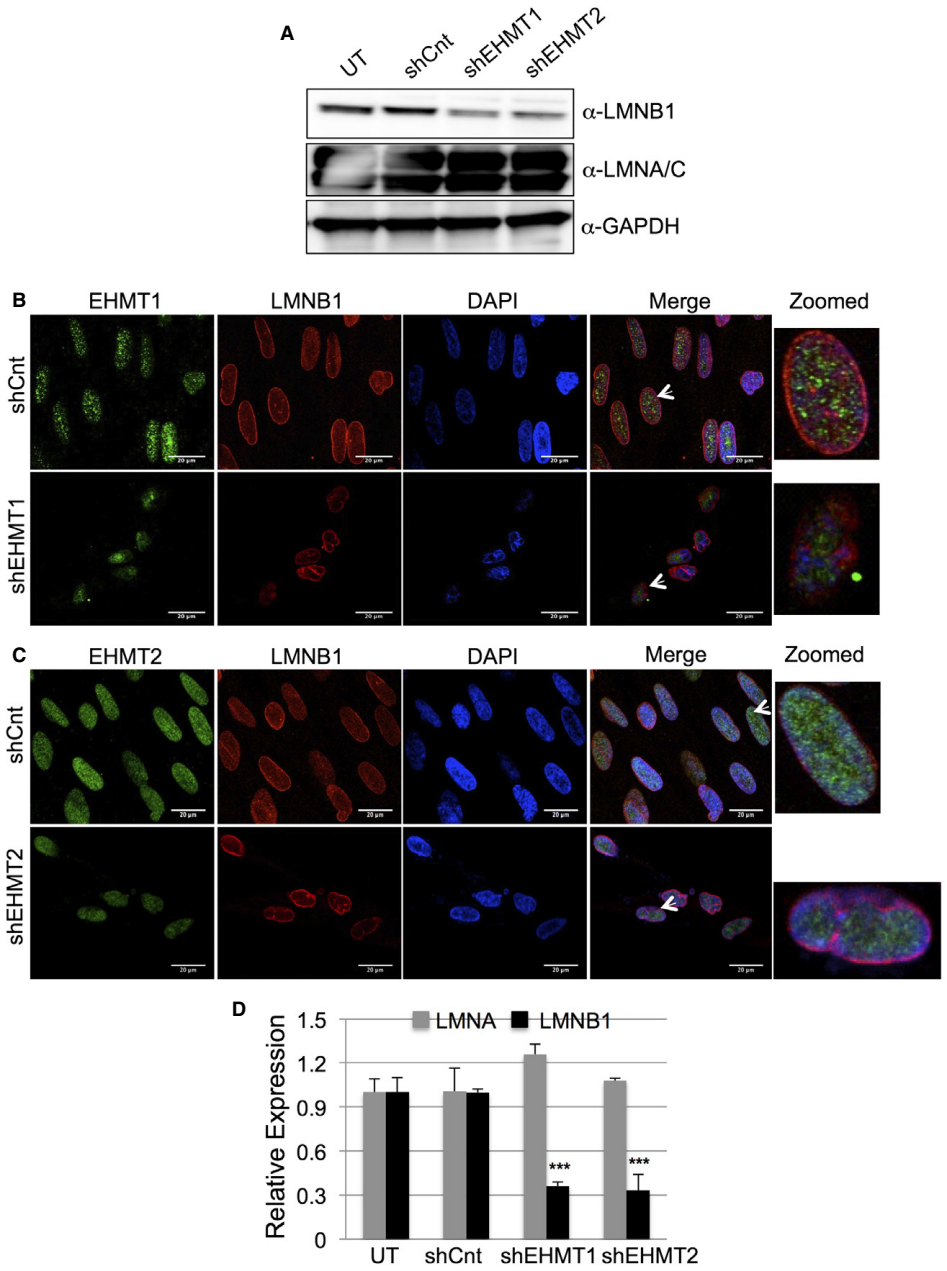
EHMT1 and EHMT2 knockdown results in reduced LMNB1 levels AWestern blot analysis for LMNB1 and LMNA/C in fetal HDFs transduced with shEHMT1 and shEHMT2 virus. Untransduced (UT) and control shRNA (shCnt) were used as controls.B, CFetal HDFs were transduced with shCnt, shEHMT1, and shEHMT2 virus. Post‐transduction cells were immunostained for EHMT1 or EHMT2 and co‐stained for NL using LMNB1 antibody. Distortion of nuclear architecture was seen upon loss of EHMT1 and EHMT2 proteins (scale bar: 20 μm). Arrows indicate the cells zoomed in the far‐right image presented.DRelative expression of LMNA and LMNB1 in fetal HDFs upon EHMT1 and EHMT2 knockdown compared to UT/shCnt cells. Data represented in the figure are from three biological replicates (*n* = 3). For LMNB1: UT versus shEHMT1, ***P < 0.0001 and UT versus shEHMT2, ***P < 0.0001 (one‐way ANOVA, post hoc test: Bonferroni's multiple comparison test). Error bars are represented as ±SEM.

To test the consequence of the loss of EHMTs on LMNB1 levels, we used shRNAs and achieved approximately 60 and 70% depletion of EHMT1 and EHMT2, respectively (Fig EV3A–C). Reduced expression of EHMT2 in shEHMT1 cells and vice‐versa indicated reciprocal stabilization of these proteins within the heteromeric complex (Fig EV3A–C). Further reduced levels of LMNB1 protein in shEHMT1 and shEHMT2 cells (Fig 3A) confirm the regulation of LMNB1 by EHMTs. Moreover, shEHMT1 and shEHMT2 cells exhibited a significant distortion of nuclear morphology (Figs 3B and C, and EV3D).

Recent studies demonstrate that the B‐type lamins are long‐lived proteins 25. Therefore, loss of such proteins is a combination of transcriptional inhibition and protein degradation. Thus, we tested if EHMT proteins regulated LMNB1 expression transcriptionally. Our data demonstrate approximately 60% loss of LMNB1 transcript upon depletion of EHMT proteins (Fig 3D). Overall, our results demonstrate that EHMT1 and 2 regulate LMNB1 expression transcriptionally and directly via post‐translational modification.

### EHMT‐mediated histone and non‐histone methylation influence peripheral heterochromatin organization

**Figure EV4:**
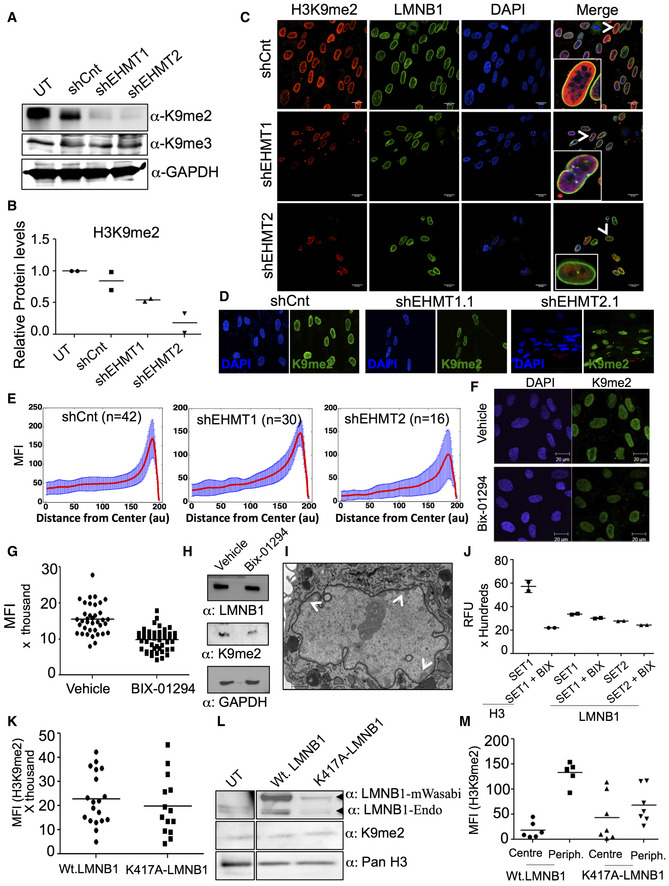
Knockdown of EHMT1 and EHMT2 reduces H3K9me2 marks Western blot analysis for H3K9me2 and H3K9me3 in UT, shCnt, shEHMT1, and shEHMT2 transduced HDFs. GAPDH was used as loading control.Quantification of H3K9me2 upon EHMT1 and EHMT2 knockdown. Each biological replicate (*n* = 2) is represented with the mean value.Immunostaining for H3K9me2 co‐stained for nuclear lamina using LMNB1 antibody in fetal HDFs transduced with shCnt, shEHMT1, and shEHMT2 virus (scale bar: 20 μm). Insets are zoomed images of cells in the merge.H3K9me2 immunostaining in fetal HDFs transduced with shEHMT1.1 (lentivirus) and shEHMT2.1 (retrovirus) (scale bar: 20 μm).The graphs show the distribution of absolute mean fluorescence intensity signal (with standard deviation) from center to the periphery of the nucleus in shCnt (nuclei = 42), shEHMT1 (nuclei = 30), or shEHMT2 (nuclei = 16) transduced HDFs. Each biological replicate (*n* = 2) is plotted as spread of technical replicates around the mean value.Immunostaining for H3K9me2 in fetal HDFs treated with or without BIX 01294 (1 μM) for 48 h. (Scale bar: 20 μm).MFI plot for H3K9me2 staining in fetal HDFs treated with or without BIX 01294 (1 μM) for 48 h. Each biological replicate (*n* = 2) is represented as spread of technical replicates around the mean value.Western blot for LMNB1 and H3K9me2 on cells treated with BIX 01294 or vehicle control. BIX 01294 treatment resulted in a reduction of H3K9me2 levels without altering LMNB1 expression.TEM image for fetal HDFs treated with BIX 01294. Unlike shEHMT1/2 that abrogated peripheral heterochromatin organization, inhibition of H3K9me2 activity by BIX 01294 did not influence peripheral heterochromatin distribution (scale bar: 1 μm)
*In vitro* methylation assay was performed using LMNB1 peptide and EHMT1‐SET or EHMT2‐SET domain in the presence or absence of BIX 01294 (*n* = 1). H3 peptide was used as a positive control (*n* = 1). Technical replicates were plotted with mean values.Quantification of MFI for H3K9me2 staining in cells transfected with Wt.LMNB1 and K417A‐LMNB1 constructs. Each biological replicate (*n* = 2) is represented as spread of technical replicates around the mean value.Western blot showing expression of endogenous LMNB1 (top panel lower band) and Wasabi tagged LMNB1 (top panel upper band). The lysates from cells transfected with Wt.LMNB1 and K417A‐LMNBI were probed with the LMNB1 antibody. The lysate was also probed with H3K9me2 antibody and H3 (loading control).MFI for H3K9me2 staining in cells transfected with Wt.LMNB1 and K417A‐LMNB1 constructs. The graphs show the absolute mean fluorescence intensity signal of H3K9me2 staining at the center and periphery of the nucleus in Wt.LMNB1 or K417A‐LMNB1 expressing HDFs. Each biological replicate (*n* = 2) is represented as spread of technical replicates around the mean value.

H3K9 methylation and LMNB1 are the critical determinants for the formation of the LADs at the NP 7, 18, 19, 20. To test the requirement of EHMT‐mediated H3K9 dimethylation in tethering peripheral heterochromatin, we depleted EHMTs and monitored the co‐localization of H3K9me2 with LMNB1. Global H3K9me2 was decreased by 50% in shEHMT1 and 80% in shEHMT2 cells (Fig EV4A and B), confirming that EHMT2 is the predominant HMTase among EHMT proteins. Though H3K9me2 is primarily localized to the NP in shCnt and shEHMT1 HDFs, there was a significant loss of enrichment of H3K9me2 in shEHMT2 cells (Figs EV4C–E and 4A).

**Figure 4:**
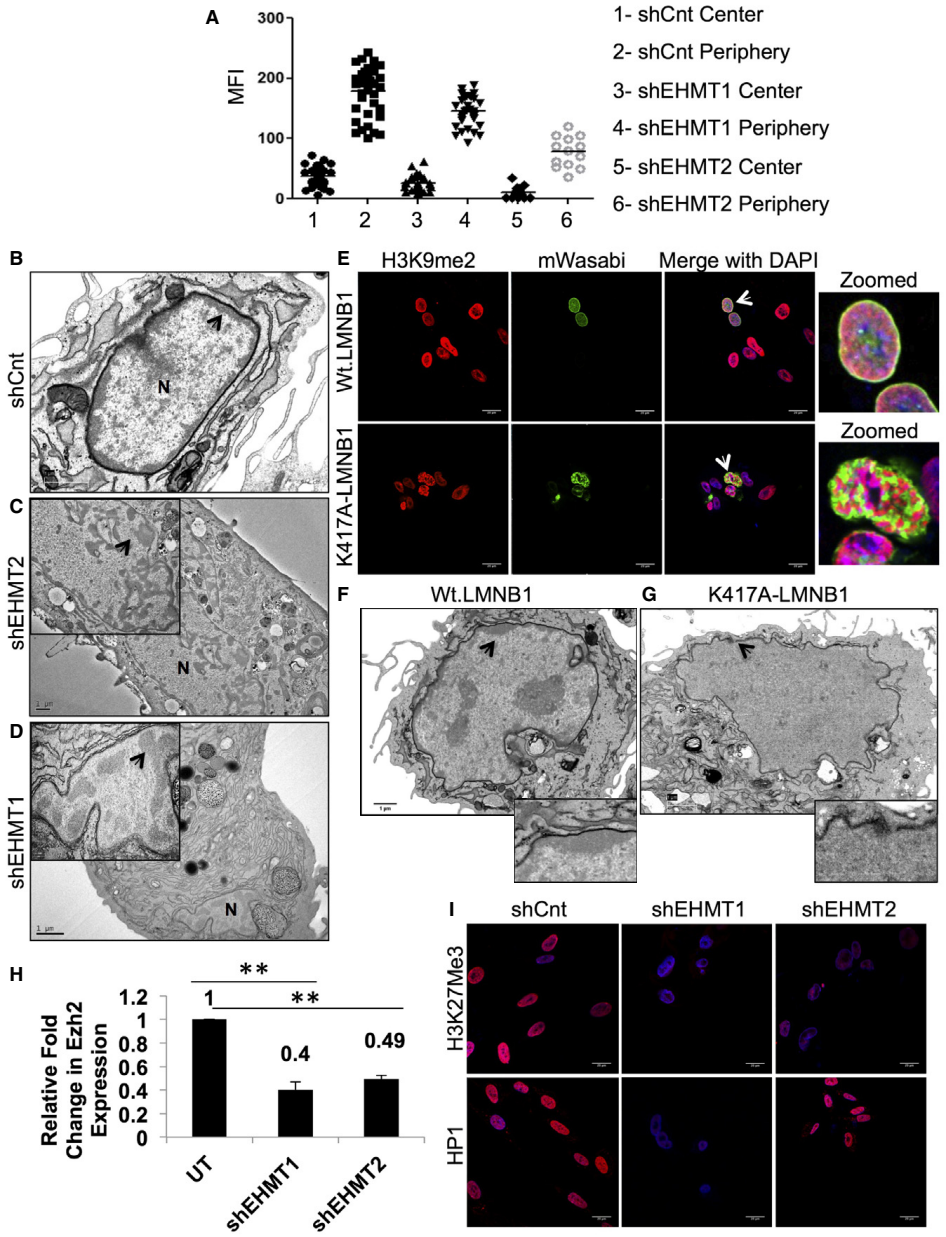
Reduction in EHMT1 and EHMT2 results in detachment of peripheral heterochromatin AThe graphs show the absolute mean fluorescence intensity signal of H3K9me2 at the center versus the periphery of the nucleus in HDFs that are transduced with shCnt, or shEHMT1, shEHMT2 transduced. Each biological replicate (*n* = 2) is represented as spread of technical replicates around the mean value.B–DFetal HDFs were transduced with shCnt, shEHMT1, and shEHMT2 virus. TEM was performed to visualize the heterochromatin in EHMT1 and EHMT2 knockdown cells. Since the nuclear size (labeled as N) was smaller in shEHMT1 and shEHMT2 cells, images of these nuclei are enlarged in zoomed boxes (scale bar: 1 μm). Peripheral heterochromatin was intact at the NP beneath the NL in control cells while this distribution was drastically altered upon knockdown of EHMT1 and EHMT2. Arrows indicate the heterochromatin regions.EImmunostaining for H3K9me2 in fetal HDFs expressing Wt.LMNB1 or K417A‐LMNB1 mutant plasmids (scale bar: 20 μm). Arrows indicate the cells zoomed in the far‐right image presented.F, GFetal HDFs transfected with Wt.LMNB1 and K417A‐LMNB1 plasmids were processed for electron microscopy. Cells transfected with Wt.LMNB1 plasmid showed intact peripheral heterochromatin while K417A‐LMNB1 transfected cells showed loss of heterochromatin with nuclear envelope breaks. Scale bar: 1 μm. Arrows indicate the zoomed area presented in the inset below.HqRT–PCR to validate expression of EZH2 in UT, shEHMT1, and shEHMT2 transduced HDFs. Three biological replicates (*n* = 3) reduced Ezh2 expression. UT versus shEHMT1 (**P = 0.0067), UT versus shEHMT2 (**P = 0.0021) (one‐sample *t*‐test, two‐tailed). Error bars are represented as ±SEM.IImmunostaining for H3K27me3 mark and HP1 protein in fetal HDFs transduced with shCnt, shEHMT1, and shEHMT2. Knockdown of EHMT1 and EHMT2 leads to a reduction in the H3K27me3 mark, which corroborates with decreased Ezh2 expression while HP1 expression was reduced only in shEHMT1 cells (scale bar: 20 μm).

Next, we performed EM to investigate the status of heterochromatin in EHMT depleted cells. shCnt transduced HDFs exhibited a layer of electron dense peripheral heterochromatin just beneath the nuclear envelope (NE) (Fig 4B). Knockdown of EHMT2 led to the partial disruption of heterochromatin from the periphery to the interior of the nucleus (Fig 4C). This result was correlated with redistribution of H3K9me2 marks toward the interior of the nucleus. In shEHMT1 HDFs, we noticed near complete detachment of peripheral heterochromatin and a distorted NE (Fig 4D). We also detected floating islands of heterochromatin in the nuclei. Interestingly, the severity of heterochromatin detachment and compromised NE integrity was unique to shEHMT1 knockdown wherein H3K9me2 activity was modestly affected.

We also looked at the effects of pharmacological inhibition of H3K9me2 on overall heterochromatin positioning and nuclear distortion. HDFs treated with BIX 01294 showed 40% less H3K9me2 staining compared to controls (Fig EV4F–H) with no changes in the LMNB1 methylation levels (Fig EV4H). Unlike EHMT depleted cells, we did not notice any significant changes in the nuclear morphology of BIX‐treated cells (Fig EV4F and I). EM imaging indicated modest changes in heterochromatin anchorage (Fig EV4I). Interestingly, addition of BIX did not influence EHMT‐mediated methylation of LMNB1 in fluorometric methyltransferase assay using LMNB1 peptide (Fig EV4J).

To obtain a clearer picture of the role of LMNB1 methylation, HDFs were transfected with either the Wt.LMNB1 or K417A‐LMNB1 construct. Overexpression of Wt. or mutant LMNB1 did not alter the levels of H3K9 dimethylation (Fig EV4K and L). Immunostaining for H3K9me2 revealed the co‐localization of H3K9me2 with LMNB1 at the NP in Wt.LMNB1 overexpressing cells (Fig 4E upper panel). In mutant‐LMNB1 expressing cells, the peripheral distribution was severely compromised and we noticed segregation of LMNB1 and H3K9me2 aggregates in the nucleoplasm (Fig 4E lower panel and Fig EV4M). EM analysis revealed that the nuclei of mutant‐LMNB1 expressing cells were devoid of peripheral heterochromatin and displayed a ruptured nuclear envelope (Fig 4F and G). These data suggest that the mislocalization of K417A LMNB1 could be responsible for the loss of peripheral distribution of chromatin. Nonetheless, our results demonstrate the significance of EHMT‐mediated LMNB1 methylation in stabilizing LMNB1 at the NP and heterochromatin organization.

**Figure EV5:**
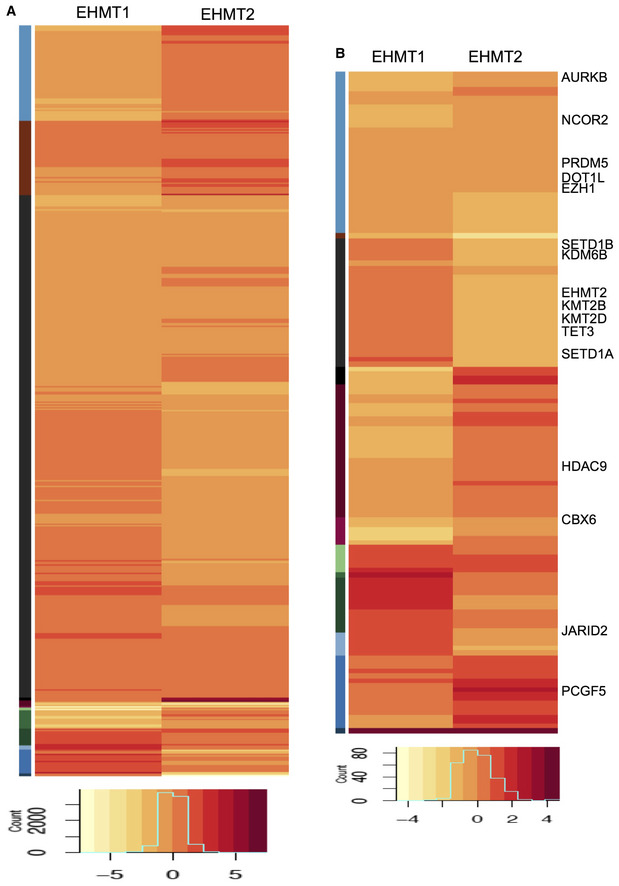
Transcriptome analysis of EHMT depleted cells A, BHeat map for differential expression of (A) all genes and (B) chromatin modifiers obtained from RNA‐Seq analysis of shEHMT1 or shEHMT2 compared to shCnt transduced HDFs. Representative genes that were altered similarly or distinctly in EHMT1 versus EHMT2 are indicated from few clusters.

To investigate the additional changes that could influence heterochromatin organization in EHMT depleted cells, we profiled gene expression changes in shEHMT1 and shEHMT2 HDFs. RNA‐Seq analysis identified overlapping and unique genes that were regulated by EHMT1 or EHMT2 (Fig EV5A, Datasets EV1 and EV2), which were enriched for genes involved in the cell cycle, homeostasis, and axon guidance, etc. (Dataset EV3). Interestingly, significantly high numbers of pathways were distinctly regulated by EHMT1 and EHMT2 (Dataset EV3). The varying degree of peripheral heterochromatin detachment in shEHMT1 versus shEHMT2 HDFs led us to investigate the number of chromatin modifiers that were altered upon EHMT depletion. While there were overlapping chromatin modifiers that changed in response to EHMT depletion, EHMT1 loss reduced expression of repressive PRC1 components (Dataset EV4). On the contrary, EHMT2 reduction predominantly influenced the expression of KDMs, SIRT6, and SETD1 proteins (Fig EV5B, Dataset EV4). Functional validation of the loss of PRC members was performed by assaying EZH2 transcript and H3K27 methyl marks. Reduced EZH2 expression and H3K27me3 staining in shEHMT1 and shEHMT2 indicate that knockdown of EHMTs affects PRC protein expression (Fig 4H and I upper panel). Interestingly, we detected the loss of HP1 only in shEHMT1 cells but not in shEHMT2 HDFs (Fig 4I, lower panel). Overall, our results revealed the commonalities and differences upon individual knockdown between two structurally similar proteins that contribute to the phenotype of peripheral heterochromatin detachment.

### Sequential loss of EHMT proteins correlates with diminished peripheral heterochromatin organization during physiological aging

The cellular phenotypes induced by EHMT1 knockdown impinge on known molecular hallmarks of cellular aging 26. These observations prompted us to survey if age‐related genes were affected in response to EHMT depletion. Comparison of RNA‐Seq profile obtained from shEHMT1 and shEHMT2 HDFs identified approximately 30% of aging‐specific genes (total 108 of 307 genes listed in the aging database) were altered in response to depletion of EHMT (Appendix Fig S1A and Dataset EV5). PCR analysis verified that genes linked to senescence, aging, and diabetes are differentially regulated upon EHMT depletion (Appendix Fig S1B and C).

**Figure 5:**
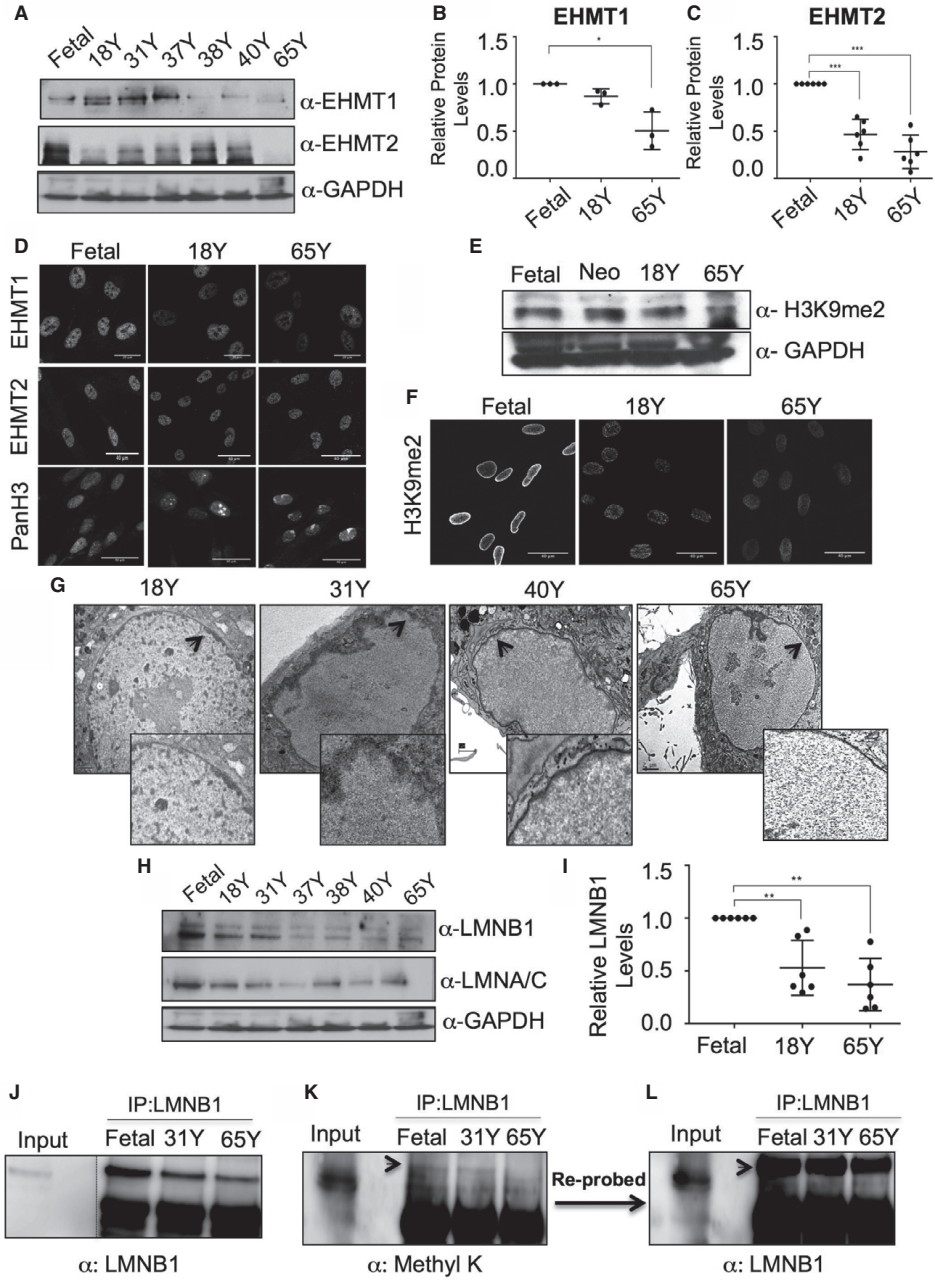
Depletion of EHMT1, EHMT2, and LMNB1 in an age‐dependent manner AWestern blot analysis for EHMT1, EHMT2, and GAPDH in HDF cell lysates derived from various age groups.BQuantification of EHMT1 protein in HDFs of indicated age groups. One sample *t*‐test (*n* = 3). For fetal versus 65, there was significant downregulation of EHMT1 protein (*P < 0.05); however, Fetal versus 18Y and Fetal versus 31Y did not show significant difference in EHMT1 levels. Error bars are represented as ±SD.CQuantification of EHMT2 protein expression in HDFs of indicated age groups. One sample *t*‐test (*n* = 6). For Fetal versus 18Y (***P = 0.0004) and Fetal versus 65Y (***P = 0.0002). Error bars are represented as ±SD.DImmunostaining for EHMT1 (scale bar: 20 μm), EHMT2 and Pan H3 (scale bar: 40 μm) in fibroblasts from indicated age groups.E, FWestern blot and immunostaining (scale bar: 40 μm) analysis for H3K9me2 in indicated age groups.GRepresentative TEM images for nuclei of 18Y, 31Y, 40Y, and 65Y HDFs. Arrows indicate peripheral heterochromatin. Inset is the zoomed version of the same images (scale bar: 1 μm).HWestern blot analysis for LMNA/C and LMNB1 and in various age group HDFs. GAPDH was used as an internal control.IQuantification of LMNB1 protein expression in HDFs of indicated age groups. One‐sample *t*‐test (*n* = 6). For Fetal versus 18Y (**P = 0.0069) and Fetal versus 65Y (**P = 0.0016). Error bars are represented as ±SD.J–LReduced levels of methylated LMNB1 during physiological aging. Nuclear extracts from indicated age groups were subjected to IP using LMNB1 antibody. IPed material was divided into halves, blot 1 (left, J) was probed with anti‐LMNB1 antibody, blot 2 was probed with anti‐Methyl‐K (middle, K) antibody. Anti‐Methyl‐K blot was reprobed with LMNB1 antibody to demonstrate the methyl band indicated is indeed LMNB1 (right, L). 80 μg of fetal HDFs derived nuclear lysate was used as input control. Dotted line indicates the spliced lane from the different exposure of the same gel (see Source data for this figure). Source data are available online for this figure.

To test whether EHMTs and H3K9me2 are indeed involved in regulating the aging process, we monitored the expression of EHMT1 and EHMT2 in HDFs derived from individuals of different age groups. As previously reported, histone methyl marks H3K27me3, H3K9me3, and HP1 are diminished in aged HDFs (Appendix Fig S1D and E). Further investigation into levels of EHMT proteins revealed a decline of EHMT1 and EHMT2 in an age‐dependent manner (Fig 5A–D). Compared to fetal cells, 18Y old HDFs exhibited decline in EHMT2 and preceded the loss of EHMT1 (Fig 5B and C). An investigation of the status of H3K9me2 methylation demonstrated its decline in aging fibroblasts (Fig 5E and F). An equally important observation was that the preferential localization of H3K9me2 was noticed at the NP in fetal HDFs, while these marks were distributed in the nucleoplasm in 18Y and 65Y HDFs (Fig 5F and Appendix Fig S2A–D). These data correlate with our observations that reduction of EHMT2 in fetal HDFs results in the re‐localization of H3K9me2 methyl marks to the nucleoplasm (Figs 4A and EV4C).

We next examined whether progressive loss of EHMTs and altered distribution of H3K9me2 impacts heterochromatin organization in aging cells. Compared to fetal cells, in 18Y and 31Y HDFs, heterochromatin was still retained at the periphery where there was a substantial loss of EHMT2 (Fig 5A and G). We also observed a redistribution of peripheral heterochromatin, which correlated with redistribution of H3K9me2 marks (Figs 4B and 5G). Further, there was a gradual loss of peripheral heterochromatin in 40Y old cells with complete depletion observed in 65Y aged nuclei (Fig 5G). Interestingly, complete loss of peripheral heterochromatin organization was correlated with loss of EHMT1 and EHMT2 in aged cells as shown in Fig 5A–C.

Since both EHMTs were downregulated during aging, we explored the regulation of EHMT1 and EHMT2 transcripts and proteins. The EHMT1 transcript was downregulated by 10% from fetal to 18Y HDFs and was further downregulated by 90% from 18Y to 65Y stage (Appendix Fig S2E). These data clearly indicated that EHMT1 expression is controlled transcriptionally during aging. On the contrary, only 30% of EHMT2 was regulated transcriptionally (20% downregulation from fetal to the adult state, with a further decline by 30% from adult to aged cells) (Appendix Fig S2E). Since a small amount of EHMT2 was regulated transcriptionally, there seemed a strong possibility of post‐translation regulation during aging. Hence, to investigate whether EHMT2 protein levels are regulated via ubiquitin–proteasome pathway (UPS), we treated 18Y and 65Y old HDFs with the proteasome inhibitor MG‐132. In 18Y old cells, EHMT2 protein levels were increased to twofold upon MG‐132 treatment, whereas no such increase was noticed in EHMT1 levels (Appendix Fig S2F and G). Interestingly, EHMT2 levels could not be rescued in 65Y HDFs (Appendix Fig S2F), indicating that the blockade of UPS activity can restore the EHMT2 degradation only in early age group and such mechanisms do not operate in aged cells wherein EHMT2 is already drastically low. Taken together, our data on the loss of EHMT1 either as a consequence of physiological aging or by forced depletion in fetal HDFs implicate EHMTs in heterochromatin organization at the NP.

Low levels of LMNB1 have been observed in senescent cells and fibroblasts derived from progeria patients 27. In this study, expression analysis of nuclear lamins in intrinsically aged cells showed decline in LMNA/C from fetal to 18Y age, and then, the protein levels remained constant in further age groups (Fig 5H). On the contrary, there was a dramatic reduction of LMNB1 starting in the 18Y age group with a significant loss at 65Y (Fig 5H and I). Diminishing levels of LMNB1 were correlated with the reduction in EHMT2 protein with drastic loss upon depletion of EHMT1 protein in 65Y cells (Fig 5A–D). This is consistent with the data in Fig 3 where we found that EHMT proteins directly regulate levels of the LMNB1 protein.

Next, we investigated whether diminishing perinuclear heterochromatin organization in aging nuclei is a result of the loss of EHMT1, EHMT2, and LMNB1 interaction. Toward this, we performed IP experiments using Fetal, 18Y, and 65Y HDF nuclear extracts. Our results revealed an association between EHMT2 and LMNB1 occurred in fetal cells and was highly diminished in adult and aged cells (Appendix Fig S2H). On the contrary, EHMT1 associated with LMNB1 in all the age groups and the interaction was reduced gradually in age‐dependent manner (Appendix Fig S2I). The complete absence of perinuclear heterochromatin in 65Y‐aged nuclei corresponded to over 80% reduction in the interaction between EHMT1 and LMNB1. These data indicated that association of EHMT1 and LMNB1 is involved in maintaining peripheral heterochromatin in aging fibroblasts.

Further, we tested the status of LMNB1 methylation during the physiological aging and found the reduced intensity of a methylated LMNB1 signal from Fetal to 31Y with virtually no methylation in 65Y‐aged cells (Fig 5J–L, Appendix Fig S2J and K). Taken together, our results indicate that the loss of peripheral heterochromatin in EHMT depleted cells or aged cells occurs due to loss of H3K9 activity coupled with the loss of LMNB1, which are critical determinants of peripheral heterochromatin anchorage.

### Overexpression of EHMT proteins rescues peripheral heterochromatin defect in aged cells

**Figure 6:**
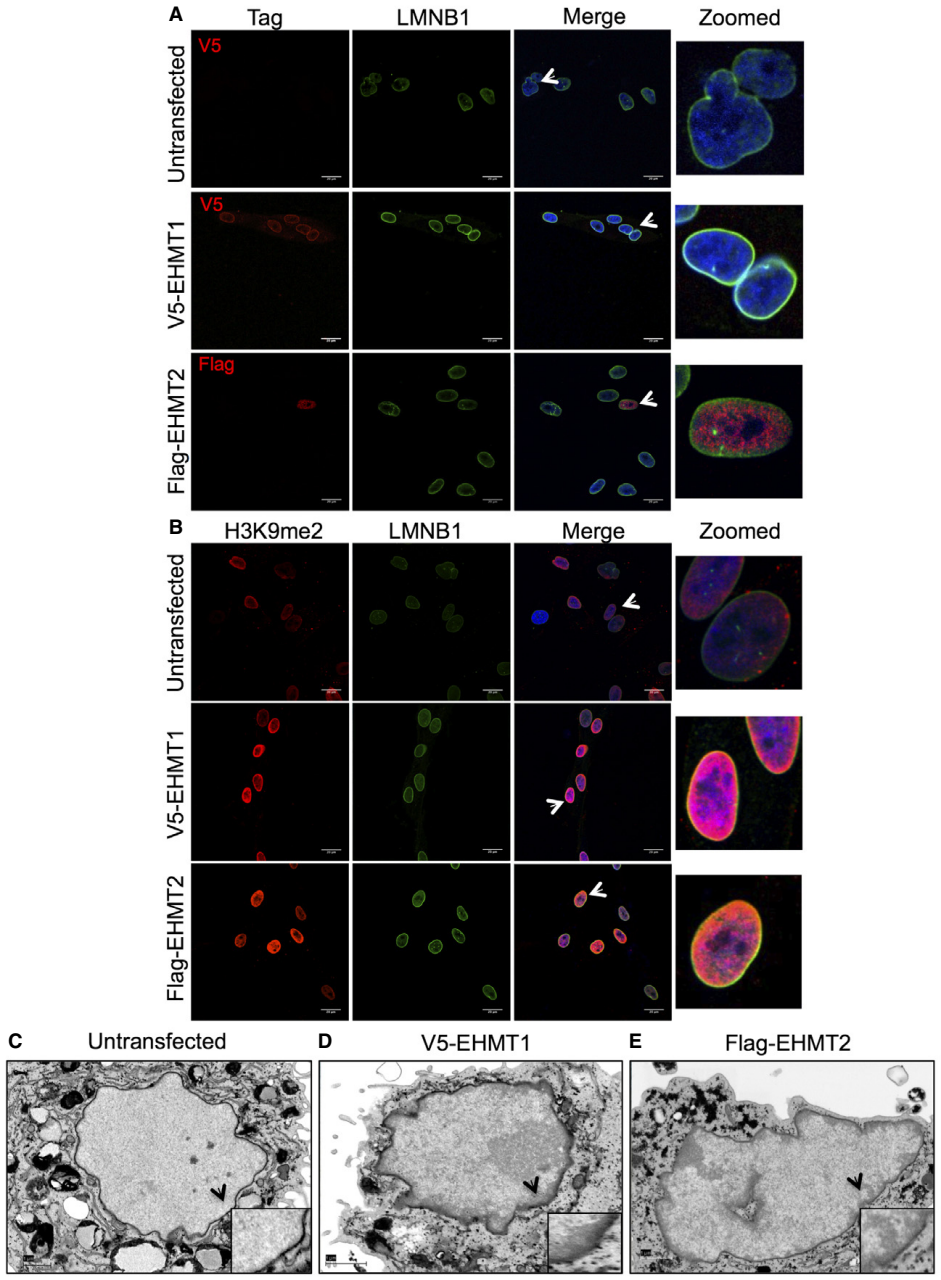
Overexpression of EHMT1 and EHMT2 restores peripheral heterochromatin organization in aged cells A, BImmunostaining using V5 or Flag antibodies along with LMNB1 in 65Y HDFs transfected with V5‐EHMT1 and Flag‐EHMT2 overexpression constructs. Immunostaining using H3K9me2 antibody along with LMNB1 in 65Y HDFs transfected with V5‐EHMT1 and Flag‐EHMT2 overexpression constructs (scale bar: 20 μm). Arrows indicate the cells zoomed in the far‐right image presented.C–E65Y old HDFs overexpressing EHMT1 and EHMT2 were processed for electron microscopy. Overexpression of EHMT1 and EHMT2 causes restoration of peripheral heterochromatin in old cells compared with control cells (scale bar: 1 μm). Arrows indicate the area zoomed and presented in the inset format.

To test whether depletion of EHMT proteins is indeed responsible for the loss of peripheral heterochromatin in aged cells, we transfected full‐length V5 tagged EHMT1 or Flag‐EHMT2 (set domain) plasmids in 65Y HDFs. Immunostaining using V5 or Flag antibodies confirmed the overexpression of EHMT1 and EHMT2 proteins (Fig 6A). EHMT1 levels enhanced the expression of LMNB1 but the Flag‐SET of EHMT2 had no effect on LMNB1 levels (Fig 6A middle and lower panel and Appendix Fig S3A and B).

We further examined H3K9me2 localization and organization of heterochromatin in EHMT1 and EHMT2 overexpressing cells. In both the cases, H3K9me2 was co‐localized with LMNB1 at the NP (Fig 6B). Consistent with this, EM imaging revealed peripherally organized heterochromatin upon EHMT1 and EHMT2 overexpression, which was completely absent in untransfected aged cells (Fig 6C–E). These results demonstrate that the reduction of EHMT proteins contributes to the loss of peripheral heterochromatin organization during aging and this defect can be reversed upon re‐expression of EHMT proteins.

To investigate the contribution of LMNB1 methylation in reversing peripheral heterochromatin tethering in aged cells, we co‐expressed wild‐type or mutant‐LMNB1 with V5‐EHMT1 or Flag‐EHMT2. As expected, Wt.LMNB1 was localized with H3K9me2 at the NP in EHMT1 overexpressing cells (Appendix Fig S3C, upper panel). Instead, H3K9me2 showed aggregated staining in the nucleoplasm and did not localize with mutant‐LMNB1 (Appendix Fig S3D and E) thereby implicating a role for methylated LMNB1 in organizing heterochromatin to the NP.

### Proliferation rate of HDFs correlate with EHMT expression

**Figure 7:**
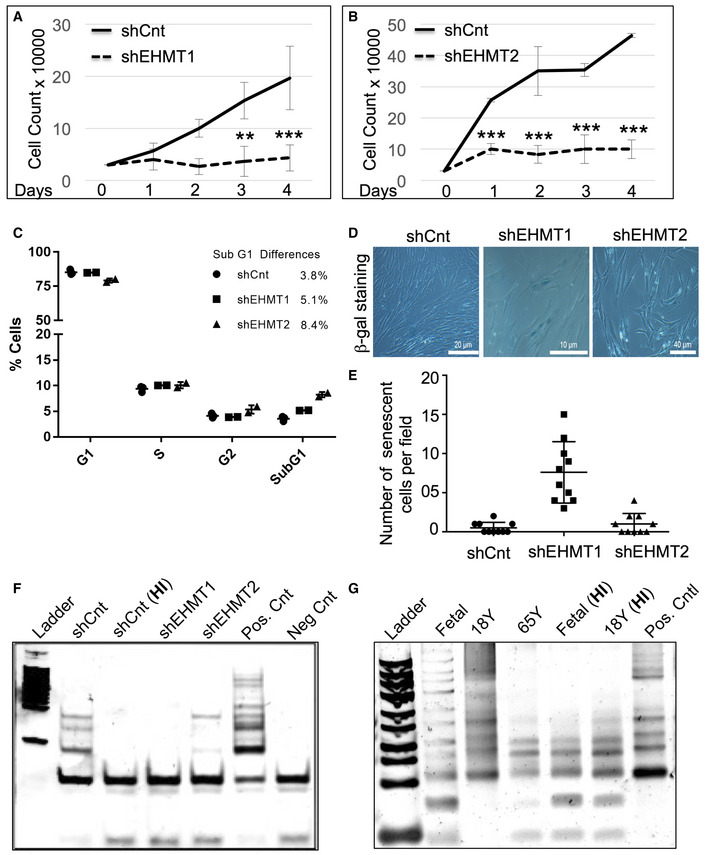
Knockdown of EHMT1 and EHMT2 leads to reduced cell proliferation and drives cells toward senescence A, BKnockdown of EHMT1 and EHMT2 reduces cell proliferation. An equal number of HDFs were seeded and transduced with shCnt, shEHMT1, and shEHMT2 virus. Forty‐eight hours post‐transduction, the number of cells in the culture was counted over a period of 4 days as indicated. (*n* = 3), for shEHMT1, Day 3: shCnt versus shEHMT1 (**P = 0.0015); Day 4: shCnt versus shEHMT1 (***P < 0.0001); for shEHMT2, Day 1: shCnt versus shEHMT2 (***P = 0.0002), Day 2: shCnt versus shEHMT2 (***P < 0.0001), Day 3: shCnt versus shEHMT2 (***P < 0.0001), Day 4: shCnt versus shEHMT2 (***P < 0.0001) (two‐way ANOVA, post hoc: Tukey's multiple comparison test). Error bars are represented as ±SD.CQuantification of cell cycle analysis. Increase in sub‐G1 population an indication of cell apoptosis upon EHMT2 knockdown. For shCnt, (*n* = 3), shEHMT1, (*n* = 2), and shEHMT2, (*n* = 2) SubG1: shCnt versus shEHMT2 (**P = 0.0011) (two‐way ANOVA, post hoc: Tukey's multiple comparison test). Error bars are represented as ±SD.D, EEqual number of HDFs was seeded and transduced with shCnt, shEHMT1, and shEHMT2 virus. β‐Galactosidase (senescence) assay was performed to monitor the cellular senescence in cultures. Number of senescent cells per field was plotted with the mean values of technical replicates (*n* = 1). Error bars are represented as ±SD.F, GTRAP assay to detect telomerase activity in indicated age groups as well as in shCnt, shEHMT1, and shEHMT2 transduced cells. Fetal, 18Y, and shCnt fibroblast lysates were heat inactivated (HI) as a negative control for the assay. Telomerase activity was reduced upon knockdown of EHMT1 and EHMT2 as well as during physiological aging from fetal to 65Y old cells.

Previous reports have demonstrated that the reduction in LMNB1 expression results in reduced proliferation and induction of premature senescence 28, 29. Therefore, we tested if the reduction of LMNB1 expression upon loss of EHMTs leads to altered proliferation. Interestingly, shEHMT1 and shEHMT2 showed a significant reduction in cell number (Fig 7A and B). Cell cycle analysis revealed a small fraction of EHMT2 transduced cells (8.4%), were in SubG1 phase compared to shCnt and shEHMT1 HDFs (Appendix Fig S4A–C and Fig 7C), indicating a small but significant apoptosis in shEHMT2 cultures. This observation is supported by transcriptome data showing the upregulation of apoptotic genes upon knockdown of EHMT2 (Datasets EV2 and EV3). shRNA‐mediated loss of EHMT proteins also induced senescence program. We observed higher number (approximately 15‐fold) of senescent cells in shEHMT1 in comparison with shEHMT2 (twofold) (Fig 7D and E).

The telomerase enzyme prevents the replicative senescence in primary fibroblast. Examining the levels of telomerase activity in shEHMT and aged cells identified a correlation between the loss of EHMT proteins with reduced telomerase activity (Fig 7F and G). Overall, our results demonstrated that the depletion of EHMT1 and EHMT2 was correlated with reduced proliferation.

## Discussion

The NL is a meshwork of lamins that constitutes the nucleoskeleton required for nuclear structure and function 8, 9, 10, 30. The NL undergoes extensive post‐translational modifications (PTMs) that are crucial for their localization to regulate a variety of biological processes 31. While uncovering the mechanism by which EHMTs organize heterochromatin, we have identified lysine methylation as a novel PTM on the nuclear localization signal (NLS) of LMNB1 that is critical for its retention at the NP and maintaining NL stability. Interestingly, this NLS motif is conserved in LMNA/C and is required for lamin–chromatin interactions 32. High‐resolution imaging of endogenous LMNA and LMNB1 demonstrated that individual homopolymers exist in close contact with each other 28. Our results showing that aggregates of mutant‐LMNB1 contain LMNA/C indicate the potential crosstalk between the two proteins via PTMs, thereby opening new avenues to explore the role of methylated LMNB1 toward the assembly of NL and its integrity. LMNA is extensively studied with several known binding partners and disease‐causing mutations 33. However, patients with laminopathies rarely exhibit a defect in the NLS sequence likely due to its lethality. In this regard, our study offers a new perspective on the less studied LMNB1 in the context of normal physiology and perhaps in laminopathies/disease.

Methylation of lysine residues facilitates a variety of functions including protein stability 34, 35, 36, 37. Altogether our data demonstrated EHMT1 and EHMT2 as upstream regulators of LMNB1 that influences its protein levels via PTMs. While EHMT2 is known to methylate a variety of non‐histone proteins 24, our study for the first time demonstrates the competency of EHMT1 to methylate non‐histone proteins and utilizing it as a mechanism to attach heterochromatin to the NP during aging. Structurally similar EHMT1/2 proteins form a heteromeric complex in mammalian cells 23 and are known to fulfill both overlapping and unique physiological roles in developing and adult animals 38. In the quest to understand the individual contributions of EHMT proteins in regulating peripheral heterochromatin, we identified that both EHMTs regulate LMNB1. However, unique molecular changes seen upon EHMT1 loss such as disruption of NE integrity High mobility Group (HMG) Proteins coupled with the loss of architectural proteins like HP1 and HMG that influence heterochromatin organization requires further investigation. Nonetheless, our studies provide a broader role for EHMTs by which it impacts the spatial distribution of the genome.

There are a number of studies demonstrating redistribution or loss of chromatin modifiers and their implications in aging 39, 40. These studies mainly focused on the consequence of the global loss of chromatin structure but none addressed the mechanisms underlying the alteration of genome architecture. Our study not only demonstrates the correlation between the expressions of EHMTs with peripheral heterochromatin organization during aging but also provides a mechanism by which EHMT regulates higher‐order chromatin structure via stabilization of the NL and architectural proteins. These results are supported by previous observations wherein defects in the sophisticated assembly of nuclear lamins along with architectural proteins result in disease or aging 40, 41, 42, 43, 44. Reorganization of heterochromatin at the NP by restoration of EHMT1 or EHMT2 in aged cells further reinforces the fact that EHMT proteins are key determinants of higher‐order chromatin organization.

Aging‐associated defects in chromatin organization exhibit a variety of functional consequences such as misregulation of gene expression via alteration of the epigenome, activation of repeat elements, and susceptibility to DNA damage 45, 46. Additionally, loss of lamins leads to altered mechano‐signaling 47. Together, these processes make aged cells stressed and also influence the stress response contributing toward reduced proliferation and enhanced senescence. Our studies revealed a direct correlation between loss of peripheral heterochromatin and reduced proliferation in shEHMT and 65Y HDFs. Taken together, we conclude that the steady loss of EHMT proteins drives normal aging in fibroblasts. It remains a mystery as to how EHMT proteins are regulated. We propose that EHMT2 degradation leads to gradual destabilization of EHMT1 in response to aging via currently unknown mechanisms. It is important to note that these results are in cultured HDFs and yet to be established if this extends in the context of tissue aging.

## Materials and Methods

### Antibodies and inhibitors

The following antibodies were used in the current study: EHMT1 (A301‐642A, Bethyl Laboratories, Rabbit polyclonal), EHMT1 (NBP1‐77400, Novus Biologicals, Rabbit polyclonal), EHMT2 (NBP2‐13948, Novus Biologicals, Rabbit polyclonal), EHMT2 (07‐551; Millipore, Rabbit polyclonal), H3K9me2 (ab1220, Abcam, Mouse monoclonal), LMNB1 (ab16048, Abcam, Rabbit polyclonal), LMNB2 (ab8983, Abcam, Mouse Monoclonal), LMNA/C (sc‐20681, Santacruz, Rabbit polyclonal), HP1‐β (ab101425, Abcam, Mouse monoclonal), H3K9me3 (ab8898, Abcam, Rabbit polyclonal), H3K27me3 (07‐449, Millipore, Rabbit polyclonal), H3 (ab1791, Abcam, Rabbit polyclonal), GAPDH (G9545, Sigma, Rabbit polyclonal), Anti‐Methyl lysine antibody (ICP0501, ImmuneChem, Rabbit polyclonal), Anti‐6X His‐tag antibody (ab9108, Abcam, Rabbit polyclonal), Anti‐GST (ab9085, Abcam, Rabbit polyclonal), Anti‐GFP (ab290, Abcam, Rabbit polyclonal), Ash2L (ab176334, Abcam, Rabbit monoclonal), p16 antibody (ab54210, Abcam), Normal Rabbit IgG (12‐370, Millipore), and Normal Mouse IgG (12‐371, Millipore). Secondary antibodies Alexa Fluor 488 Goat anti‐mouse (A‐11001), Alexa Fluor 488 Goat anti‐rabbit (A‐11008), and Alexa Fluor 568 Goat anti‐rabbit (A‐11011) were purchased from Thermo Fisher Scientific while secondary antibodies Goat anti‐mouse HRP (172‐1011) and Goat anti‐rabbit HRP (170‐8241) were from purchased Bio‐Rad. MG132 (C2211) and BIX 01294 (B9311) inhibitors were purchased from Sigma‐Aldrich.

### Cell lines

Fetal (2300), Neonatal (2310) 18Y (2320) old HDFs were purchased from ScienCell, and 38Y old HDFs (CC‐2511) were purchased from Lonza while 19Y (C‐013‐5C), 31Y (C‐013‐5C), 37Y (CC‐2511), 40Y (C‐013‐5C) and 65Y (A11634) old HDFs were purchased from Thermo Fisher Scientific. HDFs obtained from individuals of different age groups were controlled for gender (female) and ethnicity (Caucasian).

### shRNA constructs and virus preparation

EHMT1 shRNA, V3LHS_36054, was purchased from Sigma. The vector was co‐transfected with psPAX2, pMDG2 in 293‐LX packaging cell line using Lipofectamine LTX (15338500, Thermo Fisher Scientific). Viral supernatants were harvested 48 h post‐transfection and concentrated using Amicon Ultra‐15 centrifugal filter units (UFC910024, Millipore). Retroviral pSMP‐Ehmt1_4 (plasmid # 36338, Addgene), pSMP‐EHMT2_4 (Plasmid # 36334, Addgene), and pSMP‐EHMT2_1 (Plasmid # 36395, Addgene) vectors were purchased from addgene. These vectors were transfected in AmphoPack‐293 cell line (631505, Clontech). Viral supernatants were harvested 48 h post‐transfection. Viral supernatant was used to transduce HDFs. V5‐EHMT1 and Flag‐EHMT2 constructs were obtained from Dr. Marjorie Brand (OHRI, Ottawa, Canada).

### Transfection

HEK293 cells were maintained in Dulbecco's modified Eagle Medium (DMEM) (10566‐016, Thermo Fisher Scientific) supplemented with 10% fetal bovine serum (FBS) (10082147, Thermo Fisher Scientific), 2 mM l‐glutamine (25030081, Thermo Fisher Scientific), and 1% nonessential amino acids (NEAA, 11140050, Thermo Fisher Scientific). At 70% confluency, HEK293 cells were transfected with pEGFPC1, pEGFP‐ANK, or pEGFP‐SET plasmids using Lipofectamine 2000 (11668019, Thermo Fisher Scientific). HDFs were maintained in DMEM supplemented with 10% FBS, 2 mM l‐glutamine, and 1% NEAA. For knockdown experiments, cells were transduced with viral particles containing shRNAs against EHMT1 or EHMT2 and incubated for 48 h. After 48‐h transduction, cells were washed with complete media. Transduced fibroblasts were further cultured for 48 h and harvested for protein and RNA extraction. For proteasome degradation pathway inhibition experiments, cells were treated with MG132 for 6 h at 10 μM concentration. For BIX 01294 experiments, cells were treated at 1 μM concentration for 48 h. Respective inhibitor‐treated cells were further processed for Western blot, immunostaining, and/or electron microscopy.

Transfections of Wt.LMNB1 and K417A‐LMNB1 were carried out in fetal HDFs using Neon transfection method (MPK10096, Thermo Fisher Scientific). Briefly, 5 × 10^5^–2 × 10^6^ cells were collected and the pellet was washed twice with PBS. Cell pellet was then resuspended in Buffer R along with 2–3 μg of respective plasmids. Cell suspension was electroporated using Neon pipette and immediately transferred to pre‐warmed media. Transfected cells were seeded on coverslips and were processed for immunostaining or electron microscopy 6 h post‐transfection. Images for Wt.LMNB1 and K417A‐LMNB1 transfected cells were acquired at the same settings. Overexpression of EHMT1 and EHMT2 was carried out by transfecting V5‐EHMT1 and Flag‐EHMT2 overexpression constructs in old HDFs using Neon transfection method (MPK10096, Thermo Fisher Scientific) as described above. Transfected cells were seeded on coverslips and were processed for immunostaining or electron microscopy 24 h post‐transfection.

### Cell growth curve

Fetal fibroblasts transduced with shCnt or shEHMT1 and shEHMT2 were independently seeded per well of a 6‐well plate, with one well each for different time points. After each time point, cells were harvested and cell count was determined. Cell count was plotted against the time points to determine the growth curve.

### SA‐β‐galactosidase assay

SA‐β‐galactosidase staining was performed using the Senescence Cells Histochemical Staining Kit (CS0030, Sigma). Cells were rinsed with PBS followed by fixing with 1× fixation buffer provided with the kit for 8 min at RT. After rinsing thrice with PBS, 0.5 ml of the staining mixture was added and incubated at 37°C without CO_2_ for 18 h. The percentage of β‐gal‐positive cells were quantified from the images taken at 10 randomly selected microscopic fields.

### Cloning

The Ankyrin and SET domains of EHMT1 were amplified from cDNA prepared using the Superscript III cDNA synthesis kit (11752‐050, Thermo Fisher Scientific) from HDFs with the help of Ankyrin (737‐1004 AA) and SET (1013‐1265 AA) domain primers. The PCR amplified EHMT1‐Ankyrin and SET products were then cloned into pEGFPC1 vector (6084‐1, Clontech) to generate the plasmid constructs, pEGFP‐ANK and pEGFP‐SET. For cloning the C‐Term of LMNB1, the cDNA from HDFs was PCR amplified using primers LMNB12F and LMNB1‐1R and cloned into pET28a+ vector between BamHI and HindIII restriction digestion sites. The identity of all plasmids was confirmed by sequencing.

Site‐directed mutagenesis was carried out at 417^th^ lysine residue in LMNB1 plasmid construct mWasabi‐LaminB1‐10 (411^th^ position in mWasabi‐LaminB1 plasmid and 417^th^ in UniProt LMNB1 sequence). mWasabi‐LaminB1‐10 was a gift from Michael Davidson (Addgene plasmid # 56507). Briefly, 200 ng of the LMNB1 plasmid construct was subjected to a standard mutagenic PCR with *Q5* High Fidelity DNA polymerase (M0491, New England Biolabs) and 25 ng of specific primers. The primers used are as follows:

EHMT1 ANK: F‐ CCGGAATTCTTC CAC CCA AAG CAG CTG TAC
R‐ AAGGAAAAAAGCGGCCGCGCTGGGCCTGTCGGG

pEGFPC1‐ANK Construct

EHMT1 SET: F‐ CCGGAATTCGGACATCGCTCGAGGCTACGAG
R‐ AAGGAAAAAAGCGGCCGCGTGCCGGCACTTGGG

pEGFPC1‐SET Construct

LMNB1‐CT: F‐ TCGCGGATCC atgtatg aagaggagat taac
R‐ GGGAAGCTT ttacataattgcacagcttcta.

The mutagenic PCR parameters were as follows: 98°C for 1 min, 18 cycles (98°C for 15 s, 70°C for 15 s, 72°C for 2 min), and 72°C for 5 min. The final reaction volume was 50 μl. The reaction product was digested with 10 U of methylation‐sensitive enzyme *DpnI* at 37°C for 1 h. (R0176, New England Biolabs). *E*. *coli* DH5‐α competent cells were transformed with the amplified products. Finally, the plasmids were purified using the Qiagen plasmid DNA purification kit. Sequence for the K417A plasmid is provided in Dataset EV6.

### Primers used for site‐directed mutagenesis

LMNB1‐K417L: GAAAGCGGGCGAGGGTTGATGTG
LMNB1‐K417L: AACCCTCGCCCGCTTTCCTCTAG.

### qPCR and PCR

Total RNA was isolated using TRIzol reagent (15596018, Thermo Fisher Scientific) as per manufacturer instructions. RNA was subjected to cDNA synthesis using Maxima First Strand cDNA Synthesis Kit (K1641, Thermo Fisher Scientific). Semi‐quantitative PCRs were performed using 2× PCR Master Mix (K0171, Thermo Fisher Scientific). Products were resolved on 1.2% agarose gels. Quantitative PCRs were performed using Maxima SYBR green qPCR master mix (2X) (K0251, Thermo Fisher Scientific). GAPDH was used as an internal control. Primer sequences used are **TCF3** F‐ GTTTGAAACGGCGAGAAGAG R‐CGGAGGCATACCTTTCACAT

**FGFR1:** F‐ GAACAGGCATGCAAGTGAGA R‐ GCTGTAGCCCTGAGGACAAG

**FOXM1:** F‐ TCCAGAGACTGCCAGAAGGT R‐AAAAAGGACTCTGGCAAGCA

**CDK2B:** F‐CGCCCACAACGACTTTATTT R‐TTCGCTTCATGGTGAGTGTC

**LMNA:** F‐AAGCAGCGTGAGTTTGAGAG R‐TGTCCAGCTTGGCAGAATAAG

**LMNB1:** F‐ TTAGCCCTGGACATGGAAATC R‐ GATACTGTCACACGGGAAGAAG

**EZH2:** F‐ TCCTCCTGAATGTACCCCCA R‐ TGCACTTACGATGTAGGAAGC

**EHMT1:** F‐ GACATCAACATCCGAGACAA R‐ GAAAGAAAGAGGACGACACAG

**EHMT2:** F‐ GCCATGCCACAAAGTCATTC R‐ CTCAGTAGCCTCATAGCCAAAC

**GAPDH:** F‐ GAAATCCCATCACCAATCTTCCAGG R‐ GCAATTGAGCCCCAGCCTTCTC.

### Preparation of whole cell extracts

HEK293 cells transfected with pEGFPC1, pEGFP‐ANK, and pEGFP‐SET constructs were centrifuged, and cell pellets were washed with ice‐cold PBS and lysed in ice‐cold RIPA buffer containing protease inhibitor cocktail (PIC) (11697498001, Sigma) per 10^6^ cells for 10 min on ice. The cell lysates were cleared by centrifugation, and the supernatant was transferred to a fresh tube. Supernatant was used for IP experiments followed by Western blotting.

### Protein Induction and purification from bacterial cells

The plasmids containing the human EHMT1‐SET domain, EHMT1 (2IGQ), and EHMT2‐SET domain (Addgene plasmid # 25504 and Addgene plasmid # 25503, respectively) were expressed in *Escherichia coli C43 (DE3)* while LMNB1‐CT was expressed in *Escherichia coli B834 (DE3)* cultured in LB medium with 50 μg/ml of kanamycin (Sigma). EHMT1 (2IGQ) (Addgene plasmid # 25504) and EHMT2 (Addgene plasmid # 25503) were a gift from Cheryl Arrowsmith. After induction, cells were harvested and resuspended in lysis buffer containing 25 mM Tris pH 8.0, 0.5 M NaCl, 2 mM l‐Dithiothreitol (DTT, M109, Amresco), 5% glycerol, 0.05% Nonidet P‐40 Substitute (M158, Amresco), 3 μg/ml of deoxyribonuclease I (DNase I, DN25, Sigma), 50 mM imidazole supplemented with complete protease inhibitor cocktail, and 0.2 mM phenyl methyl sulfonyl fluoride (PMSF, 93482, Sigma). Cells were lysed by sonication using Vibra Cell Sonicator (Sonics & Materials Inc.). The crude extract was cleared, and the supernatant was incubated overnight with 2 ml Ni‐NTA Agarose resin pre‐washed with the lysis buffer described above. The resin was packed into Econo‐Column (738‐0014, Bio‐Rad). The column was washed, and 6x His tagged protein was eluted from the resin in protein elution buffer supplemented with 250 and 500 mM imidazole. The purity of protein was assessed by SDS–PAGE. The purified protein was concentrated, buffer exchanged, and protein dialysis was performed using Amicon Ultra‐4 centrifugal concentrators (UFC801008, Millipore,) with a molecular weight cutoff of 10 kDa, and the final concentration was estimated using the Bradford protein assay (5000006, Bio‐Rad). The protein was also subjected to mass spectrometry to assess its purity and molecular weight. In‐gel digestion for mass spectrometry analysis revealed a Mascot score of 2354.46 for 6X His EHMT1‐SET.

### Protein–protein interaction assays

For interaction assays, Ni‐NTA beads pre‐washed with IP100 buffer (25 mM Tris pH 7.5, 100 mM KCl, 5 mM MgCl_2_, 10% glycerol, 0.1% NP‐40, and 200 μM PMSF) were incubated with 300–500 ng of 6X His‐EHMT1‐SET. The beads were washed twice with IP100 buffer followed by two washes with Flag buffer (20 mM HEPES, 150 mM KCl, 2 mM MgCl_2_, 0.2 mM EDTA pH 8.0, 15% glycerol, 0.1% NP‐40, and 200 μM PMSF) both supplemented with 100 mM imidazole. The beads were then incubated with LMNB1‐GST (H00004001‐P01, Novus Biologicals) or GST alone (negative control) followed by washes with IP100 buffer and Flag buffer as mentioned above. The bead‐bound proteins were eluted and subjected to Western blotting with anti‐GST and anti‐6x His antibodies.

### Methyltransferase assay

#### Detection by Western blot

LMNB1‐CT and EHMT1‐SET/EHMT2‐SET were incubated along with 50 μM SAM in methyltransferase assay buffer in a 50 μl reaction at RT for 1 h. 12 μl of 4x‐SDS–PAGE loading dye was added to all the tubes to stop the reaction; samples were heated at 95°C for 8 min and resolved by 10% SDS–PAGE. The reaction was split into two, one was used for Western blot to probe with Anti‐Methyl‐K antibody and the other half was used to stain with Coomassie Blue stain (Coomassie Brilliant Blue R‐250, Amresco).

#### Detection by fluorescence

The methyltransferase assay was performed as per manufacturer's instructions (ADI‐907‐032, Enzo Life Sciences). Sequences for LMNB1 peptides used for the assay in Fig 2E are LMNB1‐RKR: SVRTTRGK**RKR**VDVEESEA; LMNB1‐RAR: SVRTTRGK**RAR**VDVEESEA.

### Immunoprecipitation (IP)

For IP experiments, cell or nuclear lysates (400 μg) prepared from HEK293 or HDFs to Dynabeads Protein A (10001D, Thermo Fisher Scientific) that were pre‐bound with 2–3 μg of indicated antibody and incubated overnight at 4°C. Beads were then washed and eluted in 2× loading dye. Eluted proteins were subjected to Western blotting with indicated antibodies.

### Sequential IP

For sequential IP, nuclear extract was added to Dynabeads Protein A (10001D, Thermo Fisher Scientific) pre‐bound with 3–4 μg of EHMT1, EHMT2, LMNB1, and Rabbit IgG antibodies overnight at 4°C. These beads were then washed with IP 100 and FLAG buffer. Primary IP elution was carried out with 0.1 M glycine pH 2.5–3 for 10 min. The elution was stopped by Tris buffer pH 9.5. The elute from EHMT1, EHMT2, LMNB1, and IgG was collected and incubated overnight at 4°C with Dynabeads Protein A pre‐bound with EHMT2, EHMT1, EHMT1, and IgG, respectively. The beads were subsequently washed with IP100 and FLAG buffer. The elution for secondary IP was carried out in sample buffer at 95°C. Next, eluted products were probed with respective antibodies in Western blot as mentioned in the Fig 1B.

### Chromatin immunoprecipitation

ChIP was performed as described previously Brand *et al* 48 with some modifications. In Brief, fetal HDFs were cross‐linked with 1% formaldehyde. Cells were lysed in buffer N containing DTT, PMSF, and 0.3% NP‐40. After isolation of nuclei, chromatin fractionation was done using 0.4 U of MNase (N5386, Sigma) at 37°C for 10 min. Reaction was stopped using MNase stop buffer without proteinase K. Simultaneously, antibodies against EHMT1, LMNB1, and Rabbit IgG were kept for binding with Dynabeads for 2 h at RT. After equilibration of beads, chromatin was added for pre‐clearing. To antibody bound beads, pre‐cleared chromatin was added and kept for IP at 4°C overnight.

Next day, beads were washed and eluted at 65°C for 5 min. Eluted product was subjected to reverse cross‐linking along with input samples, first with RNAse A at 65°C overnight and then with proteinase K at 42°C for 2 h. After reverse cross‐linking, DNA purification was performed using phenol–chloroform–isoamyl alcohol extraction method. The amount of DNA was quantitated using Qubit fluorometer (Thermo Fisher Scientific).

### ChIP‐seq library preparation

ChIP DNA was subjected to library preparation using TruSeq ChIP sample preparation kit from Illumina (IP‐202‐1012). Briefly, ChIP samples were processed for end repair to generate blunt ends using end repair mix. A single “A” nucleotide was added to the 3’ ends of the blunt fragments to prevent them from ligating to one another during the adapter ligation reaction. In the next step, indexing adapters were ligated to the ends of the DNA fragments. The ligated products were purified on a 2% agarose gel, and narrow 250–300 bp size range of DNA fragments were selected for ChIP library construction appropriate for cluster generation. In the last step, DNA fragments with adapter molecules on both ends were enriched using PCR. To verify the size and quality of library, QC was done on high‐sensitivity bioanalyzer chips from Agilent and the concentration was measured using Qubit dsDNA HS assay kit (Q32851, Thermo Fisher Scientific). After passing QC, samples were sequenced 75 paired end (PE) on NextSeq Illumina platform. Genotypic Technology Pvt. LTD. Bengaluru, India, performed the sequencing.

### ChIP‐seq analyses

Alignment of ChIP‐seq derived short reads to the human reference genome (UCSC hg19) was done using Bowtie2 short read aligner 49 with default parameters. Subsequently, aligned ChIP‐seq reads from two replicates were merged. Peak calling was done for each sample with their respective control using MACS 1.4 algorithm 50. The following parameters deviated from their default value: ‐ effective genome size = 2.70e+09, bandwidth = 300, model fold = 10, 30, *P*‐value cutoff = 1.00e‐03.

In order to identify regions enriched for EHMT1 and LMNB1, we employed a two‐step approach, first total peak counts for EHMT1 and LMNB1 were calculated in 1 MB window for all the chromosomes. Next, ratio of total peak count over expected peak count (total peaks from a chromosome divided by total 1 MB window for the same chromosome) was calculated for each 1 MB window. Raw data have been deposited in NCBI {SRP110335 (PRJNA391761)}.

### RNA‐Seq and data analysis

Fetal HDFs transduced with shEHMT1, shEHMT2, or shCnt were harvested, and RNA was extracted by TRIzol method. RNA concentrations were estimated using Qubit fluorometer, and quality was assessed using Bioanalyzer. After passing the QC, samples were subjected for library preparation and QC for the same. Samples were sequenced at Genotypic Technology Pvt. LTD. Bengaluru, India.

We obtained ~30–45 million reads from EHMT1 and EHMT2 knockdown samples. From the sequencing reads, adapters were trimmed using Trimmomatic program 51. The reference‐based transcriptome assembly algorithms TopHat v2.1.0 52 and Cufflinks v2.2.1 53 pipeline were used to assemble the transcripts with hg19 genome/transcriptome (http://genome.ucsc.edu/) 54 as reference annotation to guide RABT assembly. Cuffdiff v2.2.1 55 was used to identify differentially expressed genes. The transcripts with adjusted *P*‐value < 0.05 and fold change > 1.5 were considered to be significantly expressed. We used GSEA 56 to identify top 100 pathways (FDR *q* value < 0.05).

To shortlist genes involved in epigenetic modifications, Epigenetic Modifiers (http://crdd.osdd.net/raghava/dbem/) 57 and Epifactors (http://epifactors.autosome.ru/description) 58 database were used. The aging‐related genes were shortlisted from GenAge (http://genomics.senescence.info/genes/allgenes.php) database 59. Significantly expressed genes from both EHMT1 and EHMT2 knockdown datasets were overlapped with above‐mentioned databases. We used customized Perl scripts for all the analysis done in this study. All the plots and statistical analysis were done using R studio (R Development Core Team, 2011) 60. Raw data have been deposited in NCBI {SRP110335 (PRJNA391761)}.

### Immunostaining

Briefly, cells were fixed with 4% paraformaldehyde (PFA, P6148, Sigma) for 10 min at room temperature (RT) and permeabilized with 0.5% Triton X‐100. The blocking was done with 5% BSA for 1 h. Antibodies mentioned in the antibodies and inhibitors section were used at desired dilution, and imaging was carried out on FV3000 Confocal Microscope (Olympus). Image analysis and extraction of raw files were done with the Fiji software 61.

### Transmission electron microscopy

For TEM sample preparation, cells of different age groups or treated with different conditions as mentioned in Results sections were trypsinized and the pellet was fixed with 2.5% glutaraldehyde and 2% sucrose at RT for 1 h. Next, fixative was removed and pellet was washed with 0.1 M phosphate buffer pH7.4. The buffer was replaced with 1% osmium tetroxide and 1.5% potassium ferrocyanide and kept at 4°C for 75 min. Cell pellet was washed with 0.1 M phosphate buffer as well as distilled water, subsequently dehydrated with gradient of ethanol followed by two changes in propylene oxide. Cell pellet was embedded gradually in Epoxy812 resin mixture (13940, EMS). Resin‐embedded pellets were allowed to polymerize at 60°C for 72 h. The blocks were trimmed; sections of 60 nm size were collected and imaged with the Tecnai G2 Spirit Bio‐TWIN Transmission Electron Microscope at National Centre for Biological Sciences (NCBS) EM facility.

### Flow cytometry

Cells were harvested and fixed in ice‐cold 70% ethanol for 30 min at 4°C. Postfixation, cells were pelleted, washed twice in PBS, resuspended in 0.5 ml PBS containing 0.2 mg/ml RNase A (EN0531, Thermo Fisher Scientific), and incubated for 30 min at 37°C. To this, Propidium Iodide (P3566, Thermo Fisher Scientific) at a concentration of 50 μg/ml was added and incubated for 10 min at 37°C. The percentage of cells in various phases of cell cycle was assessed by flow cytometry (BD FACSVerse) and analyzed using the FlowJo software.

### Telomerase assay

Telomerase activity was detected using the PCR‐based Telomeric Repeat Amplification Protocol (TRAP) assay kit (Millipore, S7700). Briefly, different age groups HDFs or cells transduced with shEHMT1 and shEHMT2 were harvested and resuspended in 1× CHAP Buffer provided with the kit. The protocol was followed as per the manufacturer's instructions.

### Quantitation for mean fluorescence intensity

For quantitation of fluorescence intensity in Figs 4A, EV2E and EV4E and M, and Appendix Fig S2A–D, MATLAB programming was used. Briefly, centroid of nuclei was determined and 200‐line scans from center to periphery of the cells were drawn. Each line was further divided into 200 points. Average intensity distribution was calculated for each nucleus by calculating mean of 200‐line scans from center to periphery. Mean fluorescence intensity (MFI) was plotted for center versus periphery of nuclei. Mean intensity profile with standard deviation for all the measured nuclei. The Script for MATLAB program is provided in Computer Code EV1.

### Statistics

The detailed statistical analysis and methods have been described in the figure legends along with the *P*‐values for respective data sets. The data are represented as mean ± SD. For statistical analysis, GraphPad Prism version 7 software was used.

## Author contributions

RAR performed experiments on lentivirus, retrovirus production, shRNA transduction, overexpression studies, PCRs, immunostaining, TRAP assays, IPs, cell growth, and cell cycle studies. AAK performed experiments on immunostaining, Western blotting, electron microscopy, IPs, ChIP, PCRs, MG132, and BIX 01294 studies, and statistical analysis. NK is responsible for cloning and purification of EHMT1 and LMNB1 truncation proteins and LMNB1 methylation studies. PK is responsible for analysis of ChIP‐Seq data. AM cloned mammalian EHMT1 truncations and performed IP and Western blot analysis. VL is responsible for bioinformatics analysis of RNA‐Seq datasets. SDK and SOR wrote program using MATLAB for the analysis of immunostaining experiments. VKK performed Western blotting, statistical analysis, and methyltransferase assays. AG provided intellectual inputs. DP supervised bioinformatics analysis of RNA and ChIP sequencing and provided intellectual inputs on the manuscript. MB and CPC provided EHMT1 and EHMT2 overexpression constructs. SR conceptualized the project, designed the experiments, and wrote the manuscript.

## Conflict of interest

The authors declare that they have no conflict of interest.

## Supporting information



Appendix

Expanded View Figures PDF

Dataset EV1

Dataset EV2

Dataset EV3

Dataset EV4

Dataset EV5

Dataset EV6

Computer Code EV1

Review Process File

Source Data for Figure 1 and 5
