# Supplementary material for: KMT1 family methyltransferases regulate heterochromatin–nuclear periphery tethering via histone and non‐histone protein methylation
Source: EMBO Rep. 2019 Mar 12;20(5):e43260. doi: 10.15252/embr.201643260 (PMC6501005; doi:10.15252/embr.201643260)

KMT1 family methyltransferases regulate tethering of heterochromatin-nuclear periphery via histone and non-histone protein methylation

Radhika Arasala Rao[1,3ξ], Alhad Ashok Ketkar[1ξ], Neelam Kedia[1], Vignesh K Krishnamoorthy[1], Vairavan Lakshmanan[2,3], Pankaj Kumar[1], Abhishek Mohanty[1], Shilpa Dilip Kumar[2], Sufi O Raja[2], Akash Gulyani[2], ChandraPrakash Chaturvedi[5], Marjorie Brand[4], Dasaradhi Palakodeti[2], Shravanti Rampalli[1*]

Table of Contents:

1. Appendix Figure Legends (Page 2-4)
2. Appendix Figures (Appendix Fig S1- Appendix Figure S4)

Appendix Figure Legends

Appendix Fig S1. Depletion of EHMTs alters the expression of longevity-associated genes.

A Heat map demonstrating differential expression of age related genes in EHMT1 and EHMT2 depleted fibroblasts. Representative genes that were altered similarly or distinctly in EHMT1 vs EHMT2 are indicated from few clusters.

B. Semi-quantitative PCRs for validation of differentially expressed genes (age related) obtained from RNA-Seq analysis in shEHMT1, shEHMT2 HDFs.

C. Validation for differential expression of candidate genes (age related) obtained from RNA-Seq analysis by qRT-PCR (n=3), UT vs FGFR1, p=0.05; UT vs CDKN2B, p= 0.0058; UT vs FOXM1, p=0.0018 One sample t-test, two tailed.

D. Immunostaining for H3K9me3, H3K27me3 and HP1 in fetal, 18Y old and 65Y old HDFs followed by confocal imaging. (Scale bar: 20μm)

E. Western blot analysis for H3K9me3 and H3K27me3 in fetal, 18Y and 65Y old HDFs.

Appendix Fig S2. Interaction of EHMTs with LMNB1 during aging.

A-C. The graphs show the distribution of absolute mean florescence intensity signal (with standard deviation) from center to the periphery of the nucleus in in fetal (nuclei=15), 18Y (nuclei =18) and 65Y old (nuceli =22) HDFs. Individual biological replicates (n=2) are plotted with mean values.

D. The graphs show the absolute mean florescence intensity signal of H3K9me2 at the center and the periphery of the nucleus in fetal, 18Y and 65Y old HDFs. Individual biological replicates (n=2) are plotted with mean values.

E Relative expression of EHMT1 and EHMT2 at the mRNA level in fetal, 18Y and 65Y old HDFs. (n=3), For EHMT1: Fetal vs 65Y, *p=0.0219; For EHMT2: Fetal vs 65Y, #p=0.0219. (Kruskal-Wallis test, post-hoc test: Dunn's multiple comparison test).

F Western blot for EHMT1 and EHMT2 in 18Y and 65Y old HDFs treated with or without proteasomal degradation inhibitor MG132 (10μM for 6 h).

G Quantification of EHMT1 and EHMT2 protein levels in 18Y old HDFs treated with or without MG132 treatment. (n=3), for 18Y EHMT1: UT vs MG132, p=0.5989, ns; For 18Y EHMT2: UT vs. MG132 (*p=0.0154). (One sample t-test, two-tailed).

H-I Cell lysates prepared from human fibroblasts of indicated age groups were subjected for IP using EHMT1/EHMT2 antibody. IPed material was analyzed by immunoblotting using LMNB1 and LMNA/C antibodies. 30 μg of fetal cell lysate was used as input control. Dotted lines indicate that different exposures were used for Input and IP of the same western blot.

J Quantification of total LMNB1 immunoprecipitated in indicated age groups. LMNB1 IPed from fetal cells was used to normalize the values with 31 and 65Y age groups. Biological replicates (n=2) were plotted with mean value.

K Quantification of methylated LMNB1 signals in LMNB1 IPed samples in indicated age groups (n=2). Methylated LMNB1 intensity signal from fetal HDFs was used to

normalize the values with 31 and 65Y age groups. Biological replicates (n=2) were plotted with mean value.

Appendix Fig S3. Co-expression of mutant LMNB1 with EHMTs aggregates H3K9me2 in the nucleoplasm.

A-B. Quantitation of LMNB1 expression in aged HDFs upon overexpression of EHMT1 and EHMT2. Overexpression of EHMT1 significantly increased the LMNB1 expression while EHMT2 transfected cells did not show any change compared to untransfected cells. Biological replicates (n=2) of V5-EHMT1 and Flag-EHMT2 expressing cells were plotted with the mean values.

C,D,E. Old HDFs expressing Wt. LMNB1 + V5-EHMT1, K417A-LMNB1 + V5-EHMT1 and K417A-LMNB1 + Flag-EHMT2 were stained with H3K9me2 antibody. Mutation at lysine 417 position of LMNB1 affects the overall distribution H3K9me2 and morphology (Scale bar: 20µm). Arrows indicate the cells zoomed in the far right image presented.

Appendix Fig S4. Physiological aging results in cell cycle arrest. **A-C** Cell cycle analysis for shCnt, shEHMT1 and shEHMT2 transduced fetal HDFs. Each biological replicate (n=2) plotted with mean values.

Rao and Ketkar et al - Appendix Fig S1

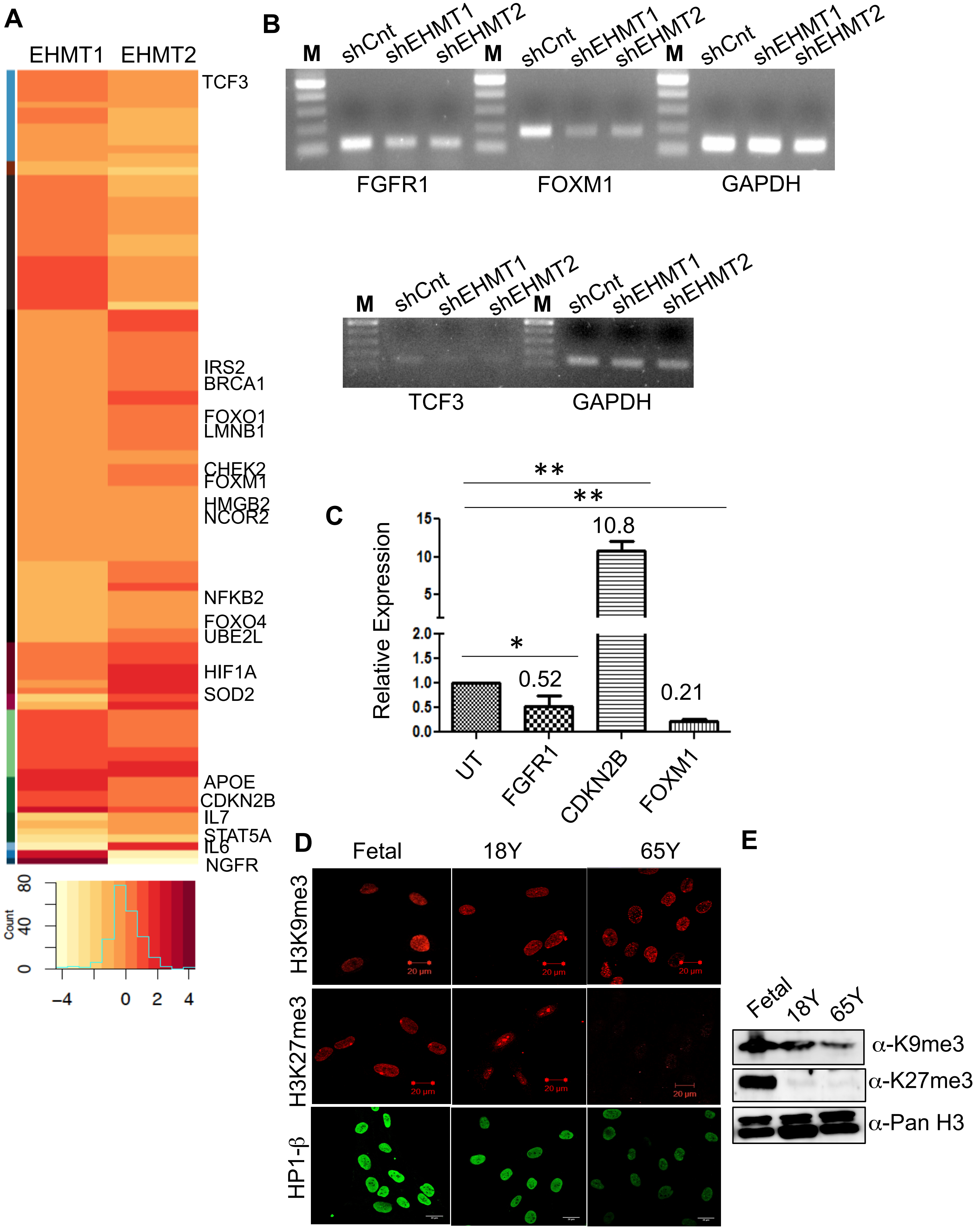

Rao and Ketkar et al - Appendix Fig S2

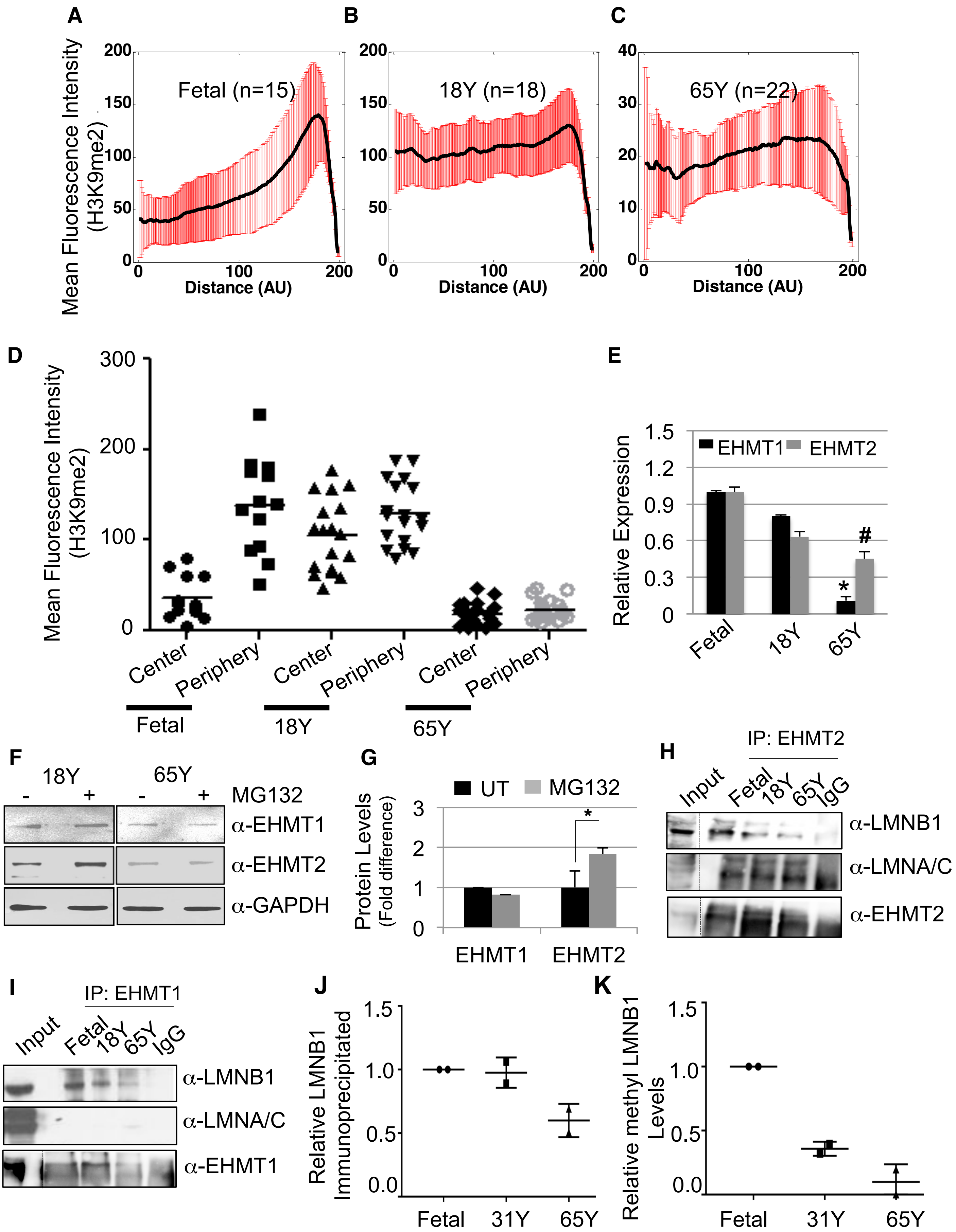

Rao and Ketkar et al - Appendix Fig S3

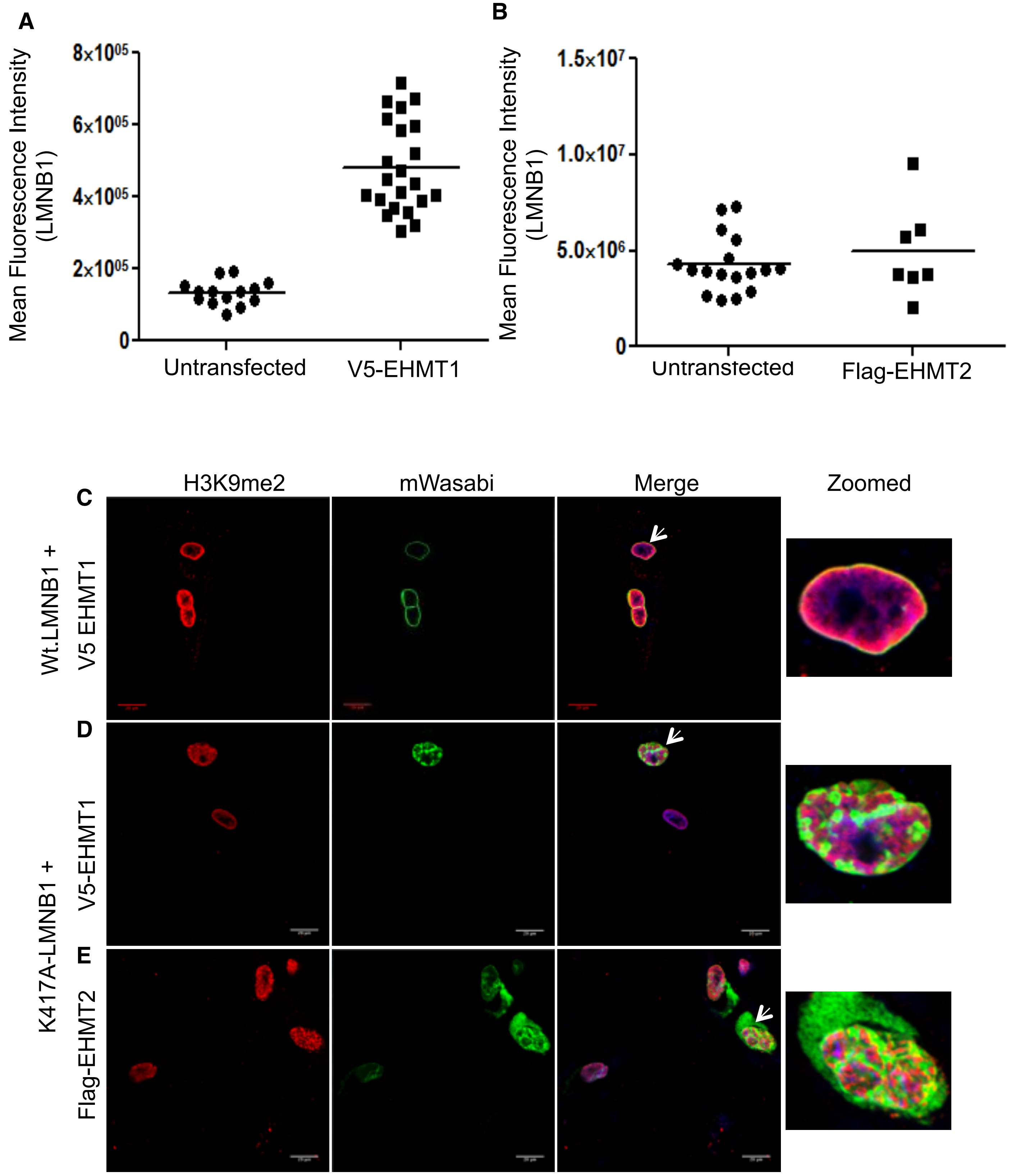

Rao and Ketkar et al - Appendix Fig S4

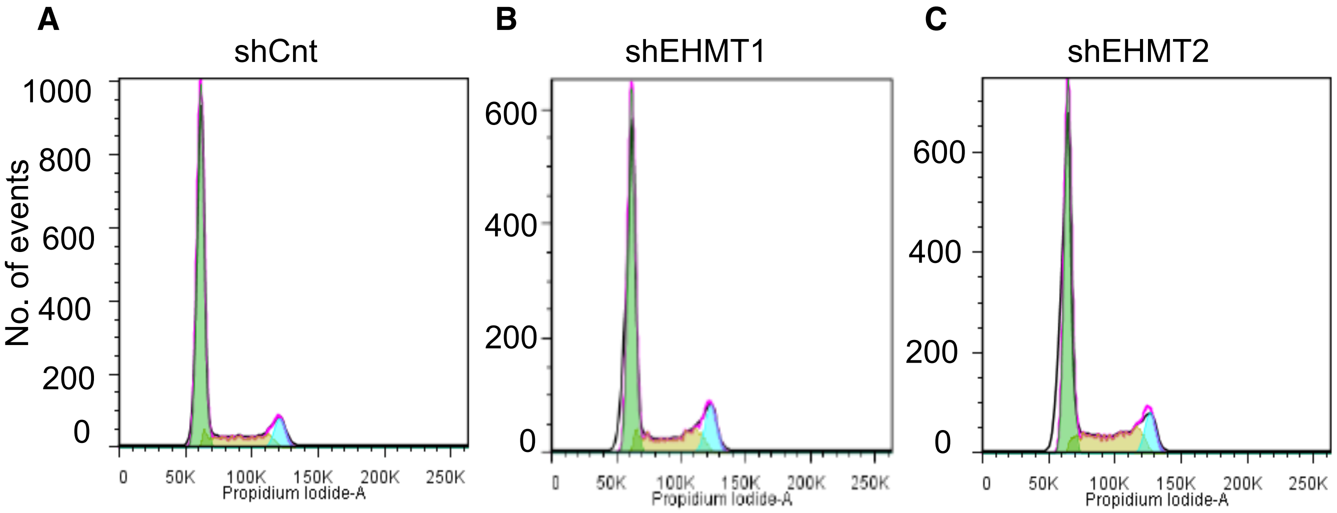

Supplement: Supplementary file 1 — Appendix [file EMBR-20-e43260-s001.pdf]
