# Supplementary material for: KMT1 family methyltransferases regulate heterochromatin–nuclear periphery tethering via histone and non‐histone protein methylation
Source: EMBO Rep. 2019 Mar 12;20(5):e43260. doi: 10.15252/embr.201643260 (PMC6501005; doi:10.15252/embr.201643260)
Supplement: Supplementary file 2 — Expanded View Figures PDF [file EMBR-20-e43260-s002.pdf]

Expanded View Figures

Figure EV1. EHMT1 interacts with LMNB1 domain and co-regulate a subset of genes.

A EHMT1 interacts with EHMT2, LMNB1, and HP1. Whole cell lysates from fetal HDFs were subjected to IP reaction using EHMT1 or LMNB1 antibody. Subsequently, Western blot was performed using the IPed material to detect EHMT1, EHMT2, LMNB1, and HP1. ASH2L did not show any interaction with EHMT1 and LMNB1 thus acted as a negative control.

B EHMT1 interacts with LMNB1 and EHMT2 via SET domain. HEK293 cells were transfected with pEGFP-Ankyrin (pEGFPC1-ANK) or pEGFPC1-SET domains of EHMT1 to determine domain-specific association with LMNB1. Cell extracts were subjected to IP using GFP-antibody, and the bound complexes were then analyzed by immunoblotting using LMNB1, EHMT2, and GFP antibodies. HEK-293 whole cell extract represents 1% input. pEGFPC1 empty vector transfected HEK293 or untransfected HEK293 cells were used as control reactions. Arrows indicate specific band.

C Coomassie-stained SDS–PAGE gel showing 6X His EHMT1-SET purified protein used for methyltransferase assays.

D–F Gene ontology (GO) analysis of EHMT1 and LMNB1 bound genes. Representative figure showing enriched GO terms for EHMT1 (D), LMNB1 (E), and EHMT1 and LMNB1 (F) co-bound genes. The length of the bar (*y*-axis) denotes total genes falling within GO term.

G Circos plot showing genome wide peak density of EHMT1 (green) and LMNB1 (red).

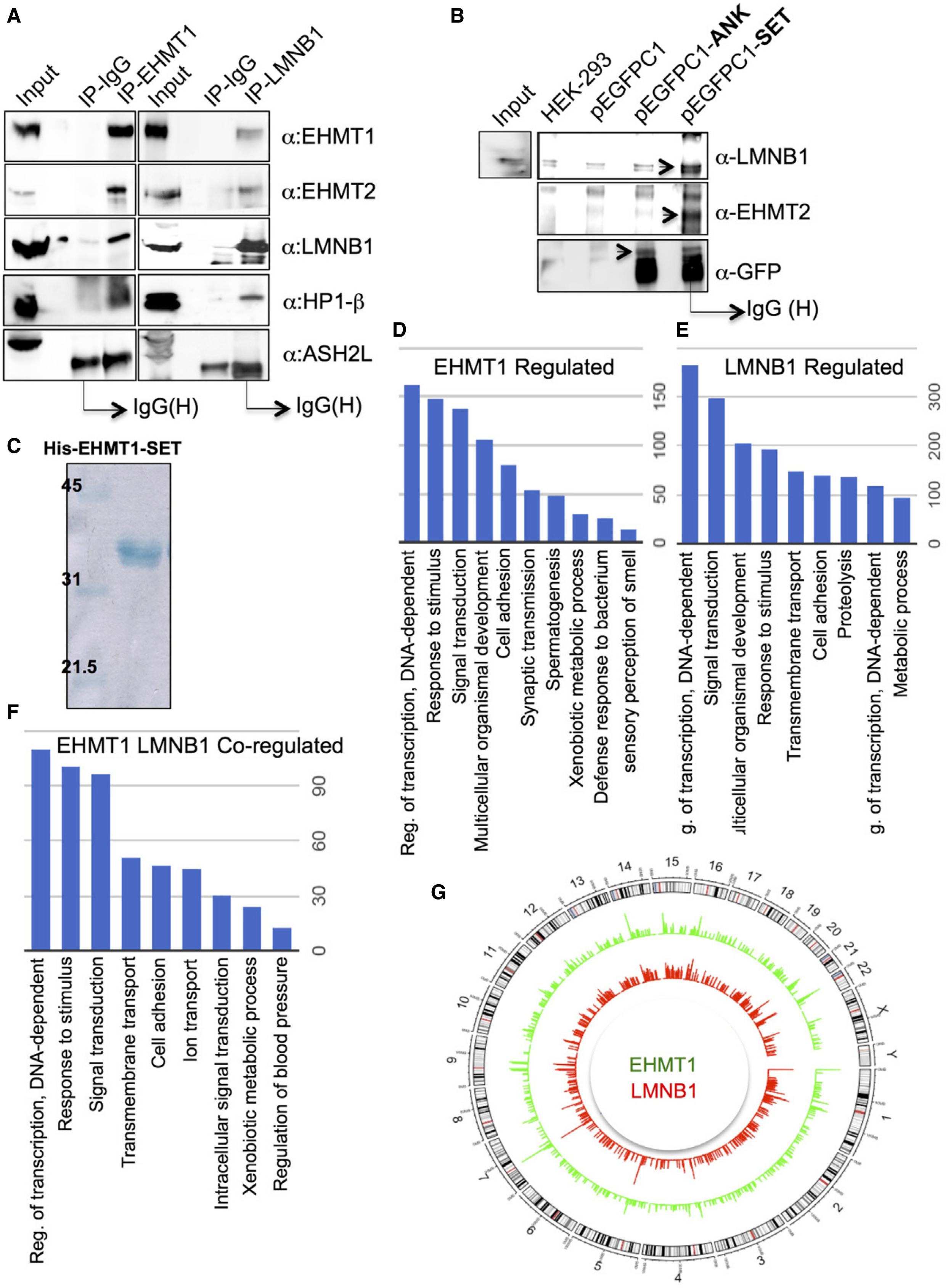

Figure EV1.

© 2019 The Authors

Figure EV2. Mutation in LMNB1 causes distortion of the nuclear architecture.

A Increasing concentrations of LMNB1-GST showed a greater degree of methylation by EHMT1-SET. Methyltransferase assay was performed using a fixed concentration of recombinant 6X His EHMT1-SET as an enzyme source and SAM as a methyl group donor. Recombinant GST-LMNB1 (4.5 ng and 9 ng) and Histone H3 peptide (10 ng) were used as substrates in the assay ($n = 2$). Histone H3 peptide was used as a positive control. The mean relative fluorescence unit (RFU) values ($n = 1$) represented in the graph were obtained after subtracting the values for controls EHMT1-SET only, SAM only, LMNB1-GST (4.5 ng) with those of the LMNB1-GST (4.5 ng and 9 ng) and Histone H3 (10 ng).

B Coomassie-stained SDS–PAGE gel showing 6X His LMNB1-CT purified protein used for methyltransferase assays.

C mWasabi expression in fetal HDFs transduced with Wt.LMNB1 and K417A-LMNB1 mutant construct (scale bar: 20 μm). Arrows indicate the cells zoomed in the far-right image presented.

D MFI for mWasabi expression in cells transfected with Wt.LMNB1 and K417A-LMNB1 mutant constructs. Each biological replicate ($n = 2$) is represented as spread of technical replicates around the mean value.

E MFI for mWasabi expression in cells transfected with Wt.LMNB1 and K417A-LMNB1 mutant constructs. MFI has been represented as center versus periphery of the nuclei. Individual biological replicates ($n = 2$) are plotted with the mean values.

F Quantitation for percentage distorted nuclei in cells transfected with K417A-LMNB1 mutant construct compared to Wt.LMNB1 construct. Individual biological replicates ($n = 2$) are plotted with the median.

G Immunostaining for LMNA/C in fetal HDFs transduced with Wt.LMNB1 and K417A-LMNB1 mutant construct (scale bar: 20 μm). Arrows indicate the cells zoomed in the far-right image presented.

© 2019 The Authors

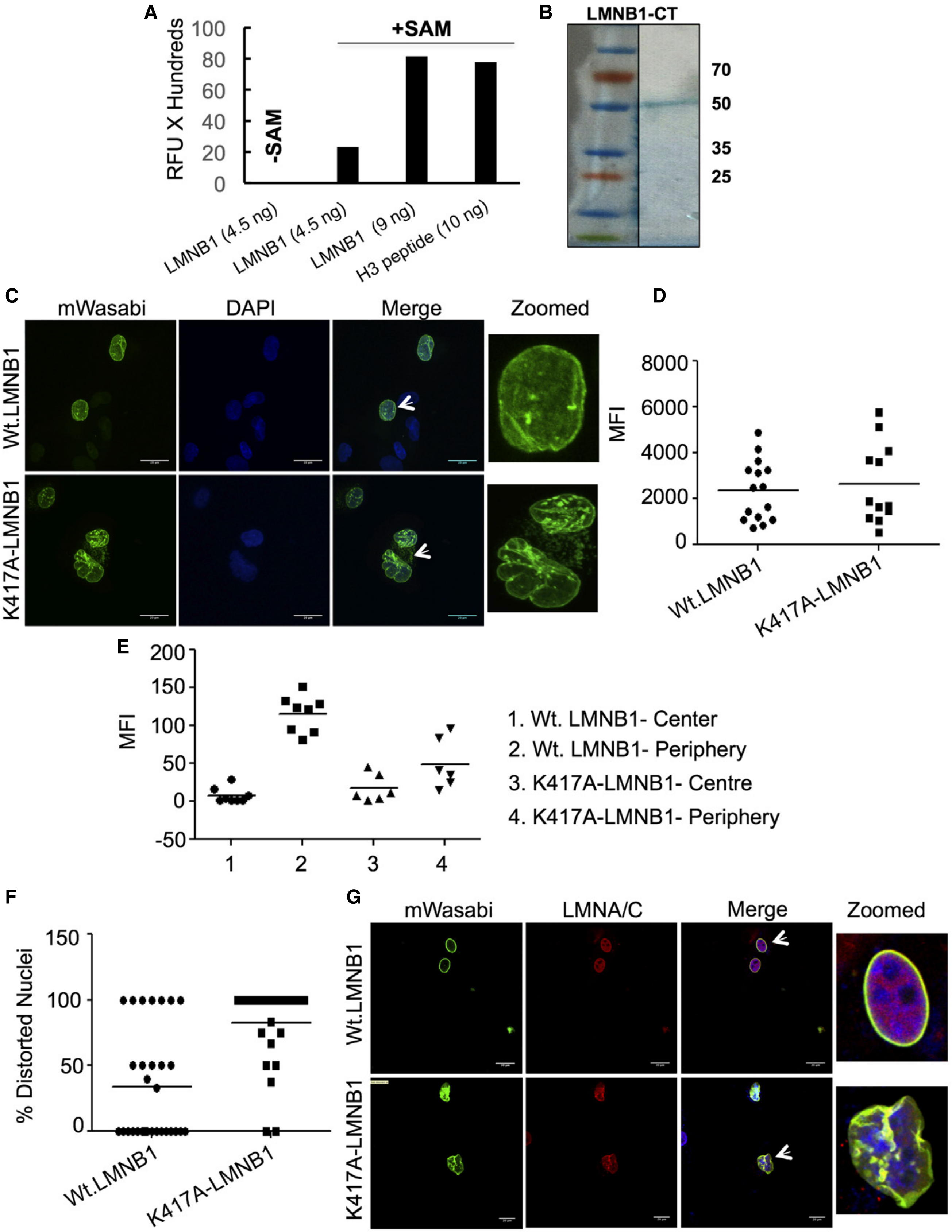

Figure EV2.

 © 2019 The Authors

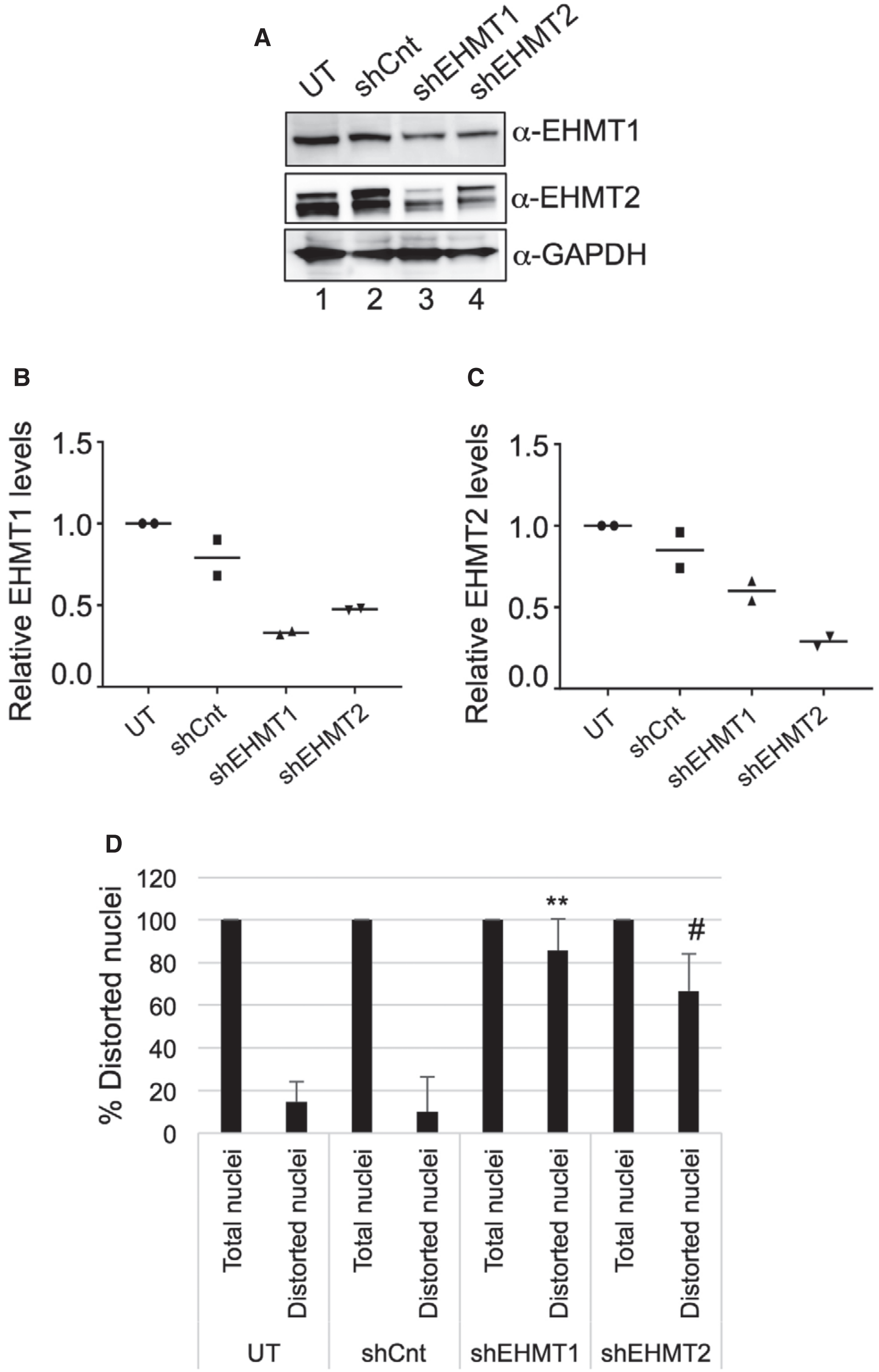

Figure EV3. Knockdown of EHMT1 and EHMT2 causes nuclear distortion.

A Western blot analysis for EHMT1 and EHMT2 in fetal HDFs upon knockdown of EHMT1 and EHMT2. GAPDH was used as a loading control.

B, C Relative protein levels of EHMT1 (B) and EHMT2 (C) upon knockdown. Each biological replicate (n = 2) is represented as spread of technical replicates around the mean value.

D Percentage of distorted nuclei increases in human fibroblasts transduced with shEHMT1 and shEHMT2 virus compared to UT or shCnt. Three biological replicates (n = 3) were used in this analysis. For UT, (nuclei = 43); shCnt, (nuclei = 23); shEHMT1, (nuclei = 18); shEHMT2, (nuclei = 20) nuclei counted. shCnt versus shEHMT1, $^{**}P$ = 0.0048; shCnt versus shEHMT2, $^{\#}P$ = 0.0153 (Kruskal–Wallis test, post hoc test: Dunn's multiple comparison test).

Figure EV4. Knockdown of EHMT1 and EHMT2 reduces H3K9me2 marks.

A Western blot analysis for H3K9me2 and H3K9me3 in UT, shCnt, shEHMT1, and shEHMT2 transduced HDFs. GAPDH was used as loading control.
B Quantification of H3K9me2 upon EHMT1 and EHMT2 knockdown. Each biological replicate (n = 2) is represented with the mean value.
C Immunostaining for H3K9me2 co-stained for nuclear lamina using LMNB1 antibody in fetal HDFs transduced with shCnt, shEHMT1, and shEHMT2 virus (scale bar: 20 μm). Insets are zoomed images of cells in the merge.
D H3K9me2 immunostaining in fetal HDFs transduced with shEHMT1.1 (lentivirus) and shEHMT2.1 (retrovirus) (scale bar: 20 μm).
E The graphs show the distribution of absolute mean fluorescence intensity signal (with standard deviation) from center to the periphery of the nucleus in shCnt (nuclei = 42), shEHMT1 (nuclei = 30), or shEHMT2 (nuclei = 16) transduced HDFs. Each biological replicate (n = 2) is plotted as spread of technical replicates around the mean value.
F Immunostaining for H3K9me2 in fetal HDFs treated with or without BIX 01294 (1 μM) for 48 h. (Scale bar: 20 μm).
G MFI plot for H3K9me2 staining in fetal HDFs treated with or without BIX 01294 (1 μM) for 48 h. Each biological replicate (n = 2) is represented as spread of technical replicates around the mean value.
H Western blot for LMNB1 and H3K9me2 on cells treated with BIX 01294 or vehicle control. BIX 01294 treatment resulted in a reduction of H3K9me2 levels without altering LMNB1 expression.
I TEM image for fetal HDFs treated with BIX 01294. Unlike shEHMT1/2 that abrogated peripheral heterochromatin organization, inhibition of H3K9me2 activity by BIX 01294 did not influence peripheral heterochromatin distribution (scale bar: 1 μm)
J *In vitro* methylation assay was performed using LMNB1 peptide and EHMT1-SET or EHMT2-SET domain in the presence or absence of BIX 01294 (n = 1). H3 peptide was used as a positive control (n = 1). Technical replicates were plotted with mean values.
K Quantification of MFI for H3K9me2 staining in cells transfected with Wt.LMNB1 and K417A-LMNB1 constructs. Each biological replicate (n = 2) is represented as spread of technical replicates around the mean value.
L Western blot showing expression of endogenous LMNB1 (top panel lower band) and Wasabi tagged LMNB1 (top panel upper band). The lysates from cells transfected with Wt.LMNB1 and K417A-LMNBI were probed with the LMNB1 antibody. The lysate was also probed with H3K9me2 antibody and H3 (loading control).
M MFI for H3K9me2 staining in cells transfected with Wt.LMNB1 and K417A-LMNB1 constructs. The graphs show the absolute mean fluorescence intensity signal of H3K9me2 staining at the center and periphery of the nucleus in Wt.LMNB1 or K417A-LMNB1 expressing HDFs. Each biological replicate (n = 2) is represented as spread of technical replicates around the mean value.

 © 2019 The Authors

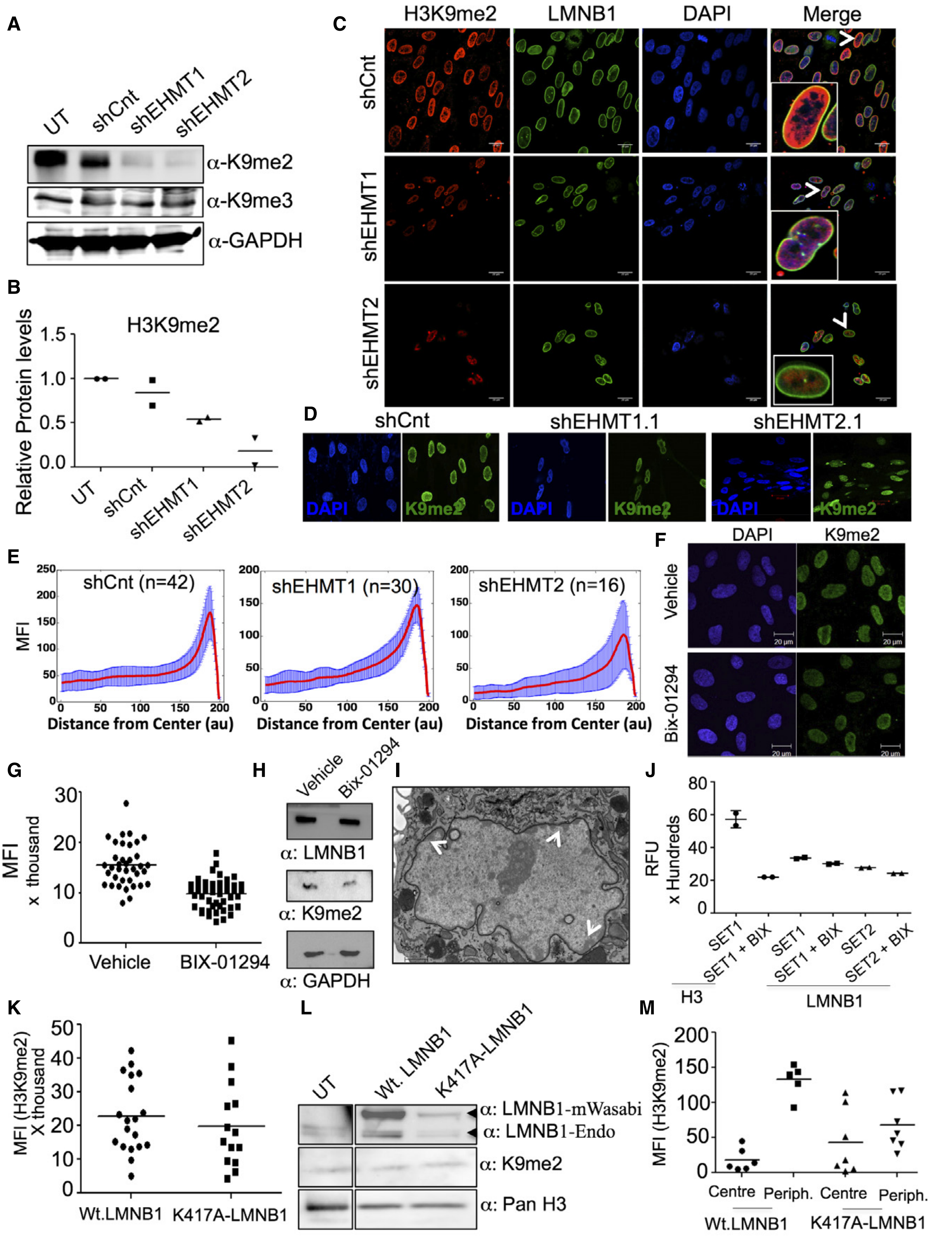
A
UT
shCnt
shEHMT1
shEHMT2
α-K9me2
α-K9me3
α-GAPDH
B
H3K9me2
Relative Protein levels
C
H3K9me2
LMNB1
DAPI
Merge
shCnt
shEHMT1
shEHMT2
D
shCnt
shEHMT1.1
shEHMT2.1
DAPI
K9me2
E
shCnt (n=42)
shEHMT1 (n=30)
shEHMT2 (n=16)
MFI
Distance from Center (au)
F
DAPI
K9me2
Vehicle
Bix-01294
G
MFI x thousand
Vehicle
BIX-01294
H
Vehicle
Bix-01294
α: LMNB1
α: K9me2
α: GAPDH
I
J
RFU x Hundreds
SET1
SET1 + BIX
SET2
SET2 + BIX
H3
LMNB1
K
MFI (H3K9me2) X thousand
Wt.LMNB1
K417A-LMNB1
L
UT
Wt. LMNB1
K417A-LMNB1
α: LMNB1-mWasabi
α: LMNB1-Endo
α: K9me2
α: Pan H3
M
MFI (H3K9me2)
Centre
Periph.
Wt.LMNB1
K417A-LMNB1

Figure EV4.

Figure EV5. Transcriptome analysis of EHMT depleted cells.

A, B Heat map for differential expression of (A) all genes and (B) chromatin modifiers obtained from RNA-Seq analysis of shEHMT1 or shEHMT2 compared to shCnt transduced HDFs. Representative genes that were altered similarly or distinctly in EHMT1 versus EHMT2 are indicated from few clusters.

© 2019 The Authors

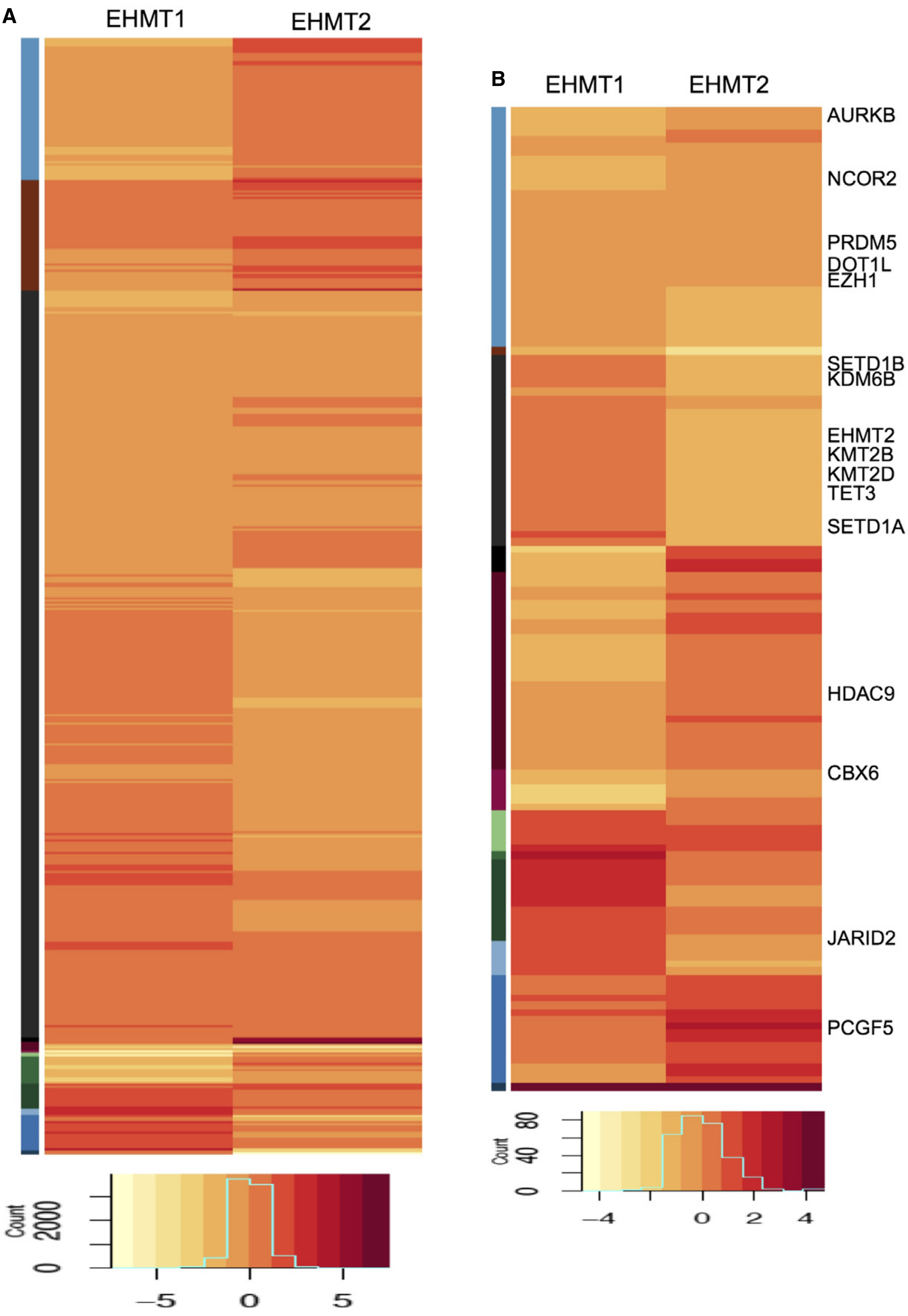
A
EHMT1
EHMT2
Count
0
2000
−5
0
5
B
EHMT1
EHMT2
AURKB
NCOR2
PRDM5
DOT1L
EZH1
SETD1B
KDM6B
EHMT2
KMT2B
KMT2D
TET3
SETD1A
HDAC9
CBX6
JARID2
PCGF5
Count
0
40
80
−4
0
2
4

Figure EV5.
