# Supplementary material for: KMT1 family methyltransferases regulate heterochromatin–nuclear periphery tethering via histone and non‐histone protein methylation
Source: EMBO Rep. 2019 Mar 12;20(5):e43260. doi: 10.15252/embr.201643260 (PMC6501005; doi:10.15252/embr.201643260)
Supplement: Supplementary file 8 — Dataset EV6 [file EMBR-20-e43260-s008.docx]

**KMT1 family methyltransferases regulate tethering of heterochromatin-nuclear periphery via histone and non-histone protein methylation**

Radhika Arasala Rao1,3, Alhad Ashok Ketkar1, Neelam Kedia1, Vignesh K Krishnamoorthy1, Vairavan Lakshmanan2,3, Pankaj Kumar1, Abhishek Mohanty1, Shilpa Dilip Kumar2, Sufi O Raja2, Akash Gulyani2, ChandraPrakash Chaturvedi5, Marjorie Brand4, Dasaradhi Palakodeti2, Shravanti Rampalli1*

**Wt. LMNB1Sequence**

ATGGCGACTGCGACCCCCGTGCCGCCGCGGATGGGCAGCCGCGCTGGCGGCCCCACCACGCCGCTGAGC C CCACGCGCCTGTCGCGGCTCCAGGAGAAGGAGGAGCTGCGCGAGCTCAATGACCGGCTGGCGGTGTACA T CGACAAGGTGCGCAGCCTGGAGACGGAGAACAGCGCGCTGCAGCTGCAGGTGACGGAGCGCGAGGAGGT G CGCGGCCGTGAGCTCACCGGCCTCAAGGCGCTCTACGAGACCGAGCTGGCCGACGCGCGACGCGCGCTC G ACGACACGGCCCGCGAGCGCGCCAAGCTGCAGATCGAGCTGGGCAAGTGCAAGGCGGAACACGACCAGC T GCTCCTCAACTATGCTAAGAAGGAATCTGATCTTAATGGCGCCCAGATCAAGCTTCGAGAATATGAAGC A GCACTGAATTCGAAAGATGCAGCTCTTGCTACTGCACTTGGTGACAAAAAAAGTTTAGAGGGAGATTTG G AGGATCTGAAGGATCAGATTGCCCAGTTGGAAGCCTCCTTAGCTGCAGCCAAAAAACAGTTAGCAGATG A AACTTTACTTAAAGTAGATTTGGAGAATCGTTGTCAGAGCCTTACTGAGGACTTGGAGTTTCGCAAAAG C ATGTATGAAGAGGAGATTAACGAGACCAGAAGGAAGCATGAAACGCGCTTGGTAGAGGTGGATTCTGGG

TGTGACAGTATCCCGAGCATCCTCAAGTCGTAGTGTACGTACAACTAGAGGAAAGCGGAAGAGGGTTGA T GTGGAAGAATCAGAGGCGAGTAGTAGTGTTAGCATCTCTCATTCCGCCTCAGCCACTGGAAATGTTTGC A TCGAAGAAATTGATGTTGATGGGAAATTTATCCGCTTGAAGAACACTTCTGAACAGGATCAACCAATGG G AGGCTGGGAGATGATCAGAAAAATTGGAGACACATCAGTCAGTTATAAATATACCTCAAGATATGTGCT G AAGGCAGGCCAGACTGTTACAATTTGGGCTGCAAACGCTGGTGTCACAGCCAGCCCCCCAACTGACCTC A TCTGGAAGAACCAGAACTCGTGGGGCACTGGCGAAGATGTGAAGGTTATATTGAAAAATTCTCAGGGAG A GGAGGTTGCTCAAAGAAGTACAGTCTTTAAAACAACCATACCTGAAGAAGAGGAGGAGGAGGAAGAAGC A GCTGGAGTGGTTGTTGAGGAAGAACTTTTCCACCAGCAGGGAACCCCAAGAGCATCCAATAGAAGCTGT

G CAATTATGTAA

**K417-LMNB1 sequence**

ATGGCGACTGCGACCCCCGTGCCGCCGCGGATGGGCAGCCGCGCTGGCGGCCCCACCACGCCGCTGAGC C CCACGCGCCTGTCGCGGCTCCAGGAGAAGGAGGAGCTGCGCGAGCTCAATGACCGGCTGGCGGTGTACA T CGACAAGGTGCGCAGCCTGGAGACGGAGAACAGCGCGCTGCAGCTGCAGGTGACGGAGCGCGAGGAGGT G CGCGGCCGTGAGCTCACCGGCCTCAAGGCGCTCTACGAGACCGAGCTGGCCGACGCGCGACGCGCGCTC G ACGACACGGCCCGCGAGCGCGCCAAGCTGCAGATCGAGCTGGGCAAGTGCAAGGCGGAACACGACCAGC T GCTCCTCAACTATGCTAAGAAGGAATCTGATCTTAATGGCGCCCAGATCAAGCTTCGAGAATATGAAGC A GCACTGAATTCGAAAGATGCAGCTCTTGCTACTGCACTTGGTGACAAAAAAAGTTTAGAGGGAGATTTG G AGGATCTGAAGGATCAGATTGCCCAGTTGGAAGCCTCCTTAGCTGCAGCCAAAAAACAGTTAGCAGATG A AACTTTACTTAAAGTAGATTTGGAGAATCGTTGTCAGAGCCTTACTGAGGACTTGGAGTTTCGCAAAAG C ATGTATGAAGAGGAGATTAACGAGACCAGAAGGAAGCATGAAACGCGCTTGGTAGAGGTGGATTCTGGG

TGTGACAGTATCCCGAGCATCCTCAAGTCGTAGTGTACGTACAACTAGAGGAAAGCGGGCGAGGGTTG AT GTGGAAGAATCAGAGGCGAGTAGTAGTGTTAGCATCTCTCATTCCGCCTCAGCCACTGGAAATGTTTGC A TCGAAGAAATTGATGTTGATGGGAAATTTATCCGCTTGAAGAACACTTCTGAACAGGATCAACCAATGG G AGGCTGGGAGATGATCAGAAAAATTGGAGACACATCAGTCAGTTATAAATATACCTCAAGATATGTGCT G AAGGCAGGCCAGACTGTTACAATTTGGGCTGCAAACGCTGGTGTCACAGCCAGCCCCCCAACTGACCTC A TCTGGAAGAACCAGAACTCGTGGGGCACTGGCGAAGATGTGAAGGTTATATTGAAAAATTCTCAGGGAG A GGAGGTTGCTCAAAGAAGTACAGTCTTTAAAACAACCATACCTGAAGAAGAGGAGGAGGAGGAAGAAGC A GCTGGAGTGGTTGTTGAGGAAGAACTTTTCCACCAGCAGGGAACCCCAAGAGCATCCAATAGAAGCTGT G CAATTATGTAA
