# Supplementary material for: KMT1 family methyltransferases regulate heterochromatin–nuclear periphery tethering via histone and non‐histone protein methylation
Source: EMBO Rep. 2019 Mar 12;20(5):e43260. doi: 10.15252/embr.201643260 (PMC6501005; doi:10.15252/embr.201643260)
Supplement: Supplementary file 9 — Computer Code EV1 [file EMBR-20-e43260-s009.docx]

**KMT1 family methyltransferases regulate tethering of heterochromatin-nuclear periphery via histone and non-histone protein methylation**

Radhika Arasala Rao1,3, Alhad Ashok Ketkar1, Neelam Kedia1, Vignesh K Krishnamoorthy1, Vairavan Lakshmanan2,3, Pankaj Kumar1, Abhishek Mohanty1, Shilpa Dilip Kumar2, Sufi O Raja2, Akash Gulyani2, ChandraPrakash Chaturvedi5, Marjorie Brand4, Dasaradhi Palakodeti2, Shravanti Rampalli1*

MATLAB Script close all;

clear all;

% [FileName,PathName,FilterIndex]=uigetfile('*.lsm','Select the cell');

% fnam = strcat(PathName,FileName);

% FileName=FileName(1:end-4);s

% BW4 = imread(fnam);

% BW_cell=BW4(:,:,2);

[FileName, FilePath]=uigetfile('*.tif',... %No inputs, lets user pick a file

'Choose tif images to import',pwd,... %"pwd" returns the folder listed as "Current Folder" in the main window

'MultiSelect','off'); %Allow only one lsm file to be chosen

if FilePath(1)==0 %If no files are chosen, break execution

disp('Error in StackSlider: No files chosen');

return

end

S.I = tiffread(strcat(FilePath,FileName));

for i=1:size(S.I,2)

I(:,:,i) = S.I(i).data;

end

BW_cell=uint8(I(:,:,1)); figure;imshow(BW_cell,[]); BW_cell_crop = imcrop((BW_cell),[]);

figure;imshow(BW_cell_crop,[]);

I_max= max(max(BW_cell_crop));

T = 0.1*I_max;

Im_bw_T = (BW_cell_crop > T);

% T = graythresh(BW_cell_crop);

%

% Im_bw = im2bw(BW_cell_crop,T-(0.9*T));

% figure;imshow(Im_bw,[])

se = strel('disk',1);

Im_bw = imdilate(Im_bw_T,se);

% figure;imshow(I2,[])

fh = imfill(Im_bw,'holes'); Im_L = bwlabeln(fh, 8);

S = regionprops(Im_L, 'Area');

Im_regProp = ismember(Im_L, find([S.Area] >= 2500));

figure;imshow(Im_regProp,[])

BW1 = edge(Im_regProp,'canny');

figure;imshow(BW1,[]);

S1 = regionprops(Im_regProp,

'Centroid','MajorAxisLength','PixelIdxList','PixelList');

centroid = S1.Centroid;

% pixels =S1.PixelList;

% figure;

% Im_regProp1=flipdim(Im_regProp,1);figure;imshow(Im_regProp1,[])

figure;[C,h]=contour(Im_regProp,[1,1]);

hold on;plot(centroid(1), centroid(2),'g+');

tic

figure;imshow(BW_cell_crop,[]);

j=1;

for i = 2:size(C,2)

xi = [C(1,i) round(centroid(1))];

yi = [C(2,i) round(centroid(2))];

[cx,cy,c,xi,yi] = improfile(BW_cell_crop,xi,yi,200);

int(:,j) = c;

j=j+1;

if mod(i,10)==0

hold on;plot(xi,yi,'r')

end j=0; toc

end

for i=1:size(mean_int,1)

flip_plot(i,:) = mean_int(size(mean_int,1)-(i-1),:);

end

figure;plot(flip_plot);
