# Supplementary material for: KMT1 family methyltransferases regulate heterochromatin–nuclear periphery tethering via histone and non‐histone protein methylation
Source: EMBO Rep. 2019 Mar 12;20(5):e43260. doi: 10.15252/embr.201643260 (PMC6501005; doi:10.15252/embr.201643260)

Source File for Figure 1B: Left Panel

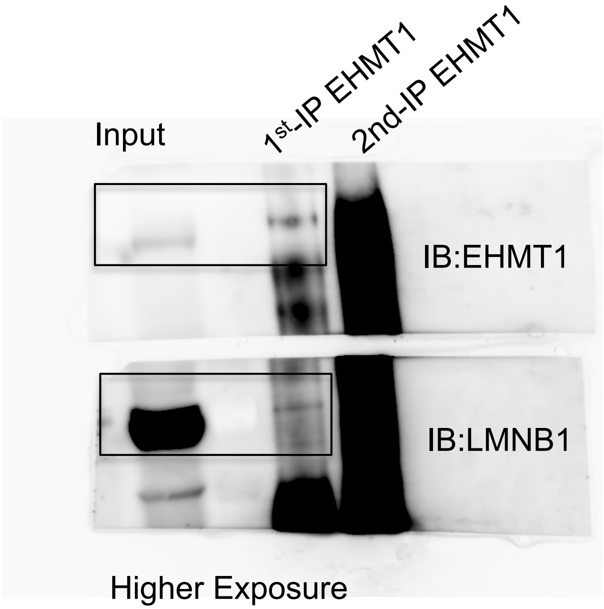

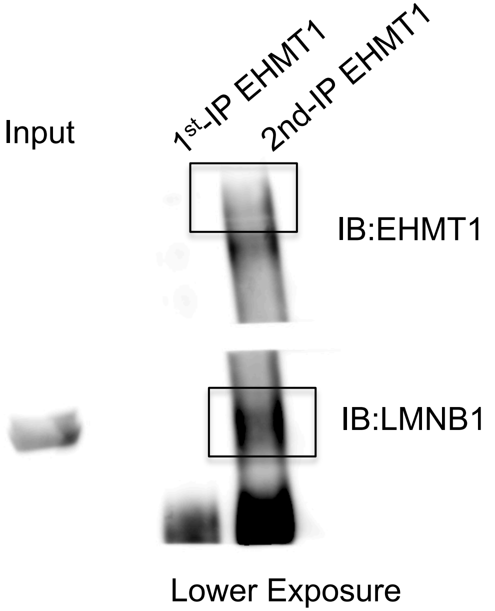

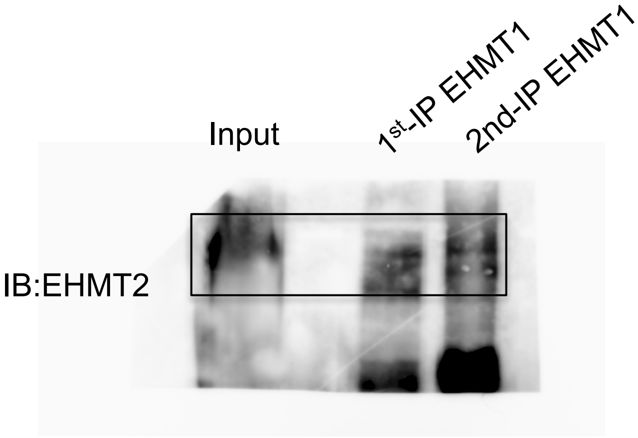

Source File for Figure 1B: Right Panel

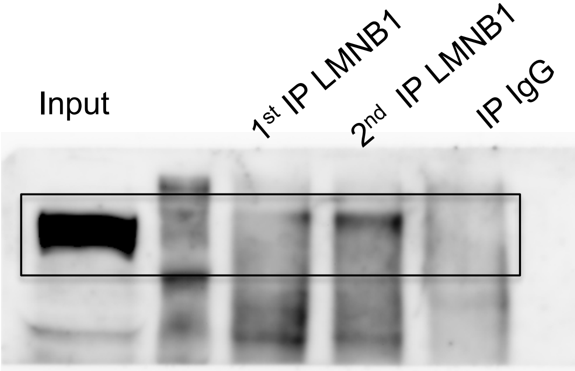

IB: EHMT1

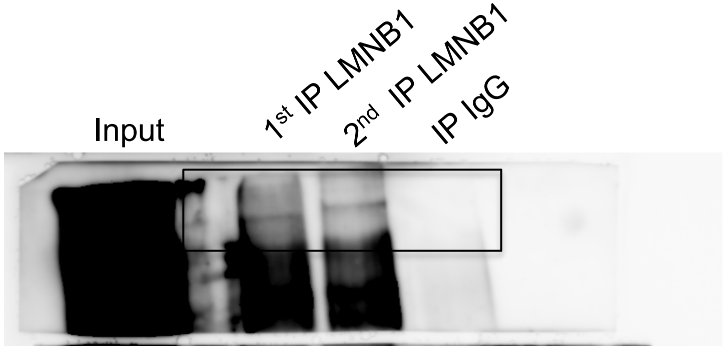

IB: EHMT2 (Higher Exposure)

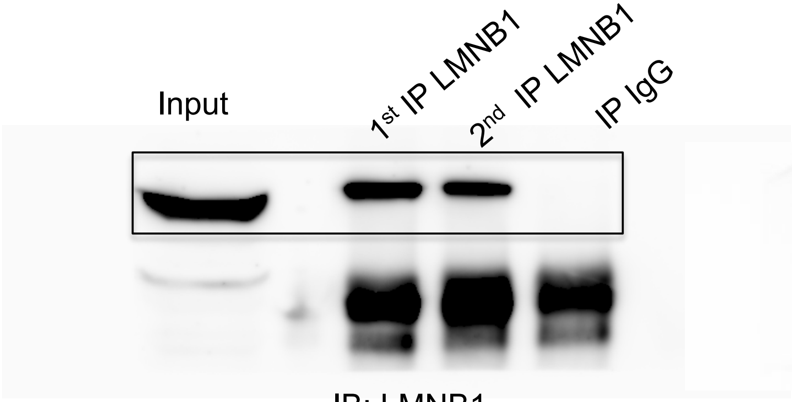

IB: LMNB1

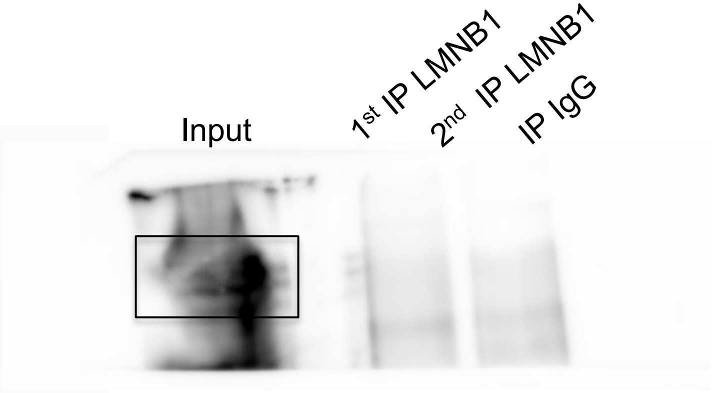

IB: EHMT2 (Lower Exposure)

Source File for Figure 5J and 5K

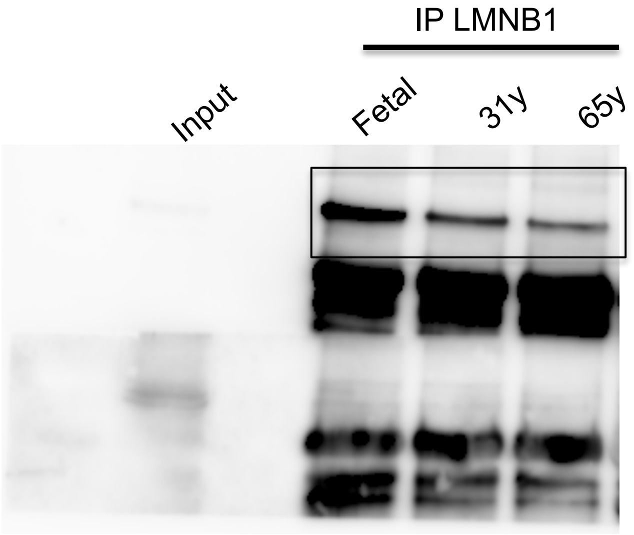

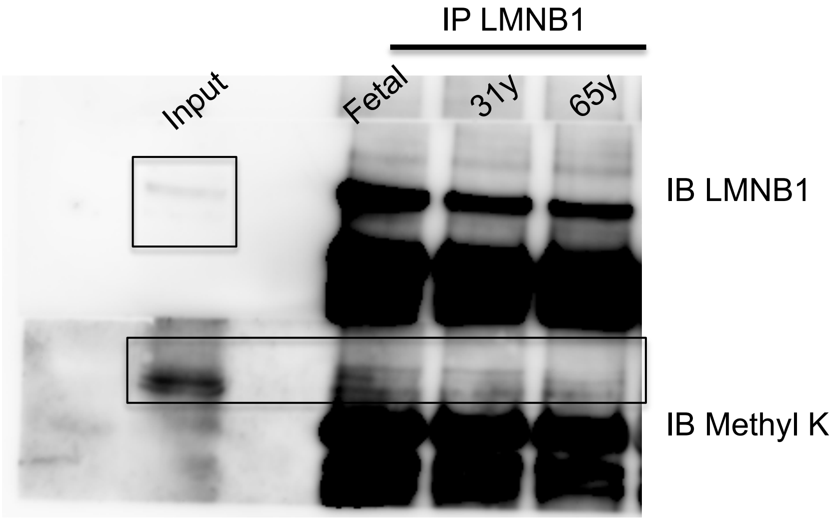

Supplement: Supplementary file 11 — Source Data for Figure 1 and 5 [file EMBR-20-e43260-s010.pdf]
